# Clinical impact of gene mutations and lesions detected by SNP-array karyotyping in acute myeloid leukemia patients in the context of gemtuzumab ozogamicin treatment: Results of the ALFA-0701 trial

**DOI:** 10.18632/oncotarget.1536

**Published:** 2014-01-20

**Authors:** Aline Renneville, Raouf Ben Abdelali, Sylvie Chevret, Olivier Nibourel, Meyling Cheok, Cécile Pautas, Rémy Duléry, Thomas Boyer, Jean-Michel Cayuela, Sandrine Hayette, Emmanuel Raffoux, Hassan Farhat, Nicolas Boissel, Christine Terre, Hervé Dombret, Sylvie Castaigne, Claude Preudhomme

**Affiliations:** ^1^ Laboratory of Hematology, Biology and Pathology Center, CHRU of Lille, Lille; ^2^ University of Lille Nord de France, Lille; ^3^ Inserm, U837, Team 3, Cancer Research Institute of Lille, Lille; ^4^ UMR-717, Inserm; Paris Diderot university, Saint-Louis Hospital, APHP, Paris; ^5^ Department of Hematology, Henri Mondor Hospital, APHP, Créteil; ^6^ Department of Hematology, Huriez Hospital, CHRU of Lille, Lille; ^7^ Laboratory of Hematology, Saint-Louis Hospital, APHP, Paris; ^8^ Laboratory of Molecular Biology and UMR5239 CNRS, Lyon Sud Hospital, Pierre-Bénite; ^9^ EA 3518, University of Paris 7; Department of Adult Hematology, Saint-Louis Hospital, APHP, Paris; ^10^ Department of Hematology, Versailles Hospital, Le Chesnay; ^11^ Laboratory of Cytogenetics, Versailles Hospital, Le Chesnay; all in France

**Keywords:** acute myeloid leukemia, SNP array lesions, gene mutations, gemtuzumab ozogamicin, prognosis

## Abstract

We recently showed that the addition of fractionated doses of gemtuzumab ozogamicin (GO) to standard chemotherapy improves clinical outcome of acute myeloid leukemia (AML) patients. In the present study, we performed mutational analysis of 11 genes (*FLT3, NPM1, CEBPA, MLL, WT1, IDH1/2, RUNX1, ASXL1, TET2, DNMT3A), EVI1* overexpression screening, and 6.0 single-nucleotide polymorphism array (SNP-A) analysis in diagnostic samples of the 278 AML patients enrolled in the ALFA-0701 trial. In cytogenetically normal (CN) AML (n = 146), 38% of the patients had at least 1 SNP-A lesion and 89% of the patients had at least 1 molecular alteration. In multivariate analysis, the independent predictors of higher cumulative incidence of relapse were unfavorable karyotype (P = 0.013) and randomization in the control arm (P = 0.007) in the whole cohort, and *MLL* partial tandem duplications (P = 0.014) and *DNMT3A* mutations (P = 0.010) in CN-AML. The independent predictors of shorter overall survival (OS) were unfavorable karyotype (P < 0.001) and SNP-A lesion(s) (P = 0.001) in the whole cohort, and SNP-A lesion(s) (P = 0.006), *DNMT3A* mutations (P = 0.042) and randomization in the control arm (P = 0.043) in CN-AML. Interestingly, CN-AML patients benefited preferentially more from GO treatment as compared to AML patients with abnormal cytogenetics (hazard ratio for death, 0.52 *versus* 1.14; test for interaction, P = 0.04). Although the interaction test was not statistically significant, the OS benefit associated with GO treatment appeared also more pronounced in *FLT3* internal tandem duplication positive than in negative patients.

## INTRODUCTION

Acute myeloid leukemia (AML) is a heterogeneous group of hematological malignancies with variable responses to therapy. Age and karyotype have been recognized as the most prominent prognostic factors in AML patients. Over the last decade, it has been reported that mutations of some genes of interest may have prognostic significance, especially in patients with cytogenetically normal (CN) AML. Prognostic impact within CN-AML has been well established for *FLT3* internal tandem duplication (*FLT3*–ITD), *NPM1* and *CEBPA* mutations. Mutations of these three genes allowed a refined prognostic classification of CN-AML and improved risk stratification in this subset of AML patients [1, 2]. With recent progress in genomic technologies, a large number of recurrent somatic mutations have been discovered in AML, including mutations in *ASXL1* [3], *TET2* [4], *IDH1/2* [5], *DNMT3A* [6, 7]*, PFH6* [8], and *BCOR* [9], thus providing new insights into the mechanisms of leukemogenesis and further evidence of the genetic complexity of AML. Retrospective studies have suggested that these novel mutations may have prognostic significance in AML, but these findings need to be confirmed and validated prospectively in clinical trial cohorts [10–12].

For years, standard induction chemotherapy in AML patients has relied on the combination of cytarabine and anthracycline, the so-called 7 + 3 regimen. We recently published the results of the ALFA-0701 Phase 3 trial, showing that the addition of fractionated doses of gemtuzumab ozogamicin (GO) during induction and consolidation may significantly improve outcome of AML patients aged 50–70 years [13]. GO is a humanized immunoconjugate targeting the CD33 antigen, a myeloid antigen expressed on the majority of AML leukemic cells. Similar positive results have been reported in two Medical Research Council (MRC) trials [14, 15], and, as in the ALFA trial, it appeared that patients with favorable or intermediate cytogenetics, including those with CN-AML, may benefit from GO, while not those with adverse karyotype. In this context, there is a growing interest in identifying molecular determinants of response to this targeted agent.

Refining conventional cytogenetics (CC) represents another promising approach to improve prognostic classification of AML, as CC detects an abnormal karyotype in only half of AML patients. Single-nucleotide polymorphism array (SNP-A)–based karyotyping has revealed previously unrecognized acquired copy number abnormalities (CNA) in AML genome [16, 17]. In addition to a high level of resolution, SNP-A allows the detection of copy-neutral loss of heterozygosity (CN-LOH), also referred to as uniparental disomy (UPD) [17]. Moreover, some studies reported that novel lesions detected by SNP-A-based karyotyping may complement CC and improve AML outcome prediction [18–20].

In the present study, we thus performed SNP-A-based karyotyping and extensive mutational analysis in the 278 AML patients enrolled on the ALFA-0701 trial. Our objectives were first to evaluate the prognostic impact of these genetic alterations on clinical outcome, particularly within the subset of CN-AML, and second to analyze potential interactions between these genetic alterations and GO treatment.

## RESULTS

### Patient characteristics at diagnosis

Baseline patient and AML characteristics are provided in Tables S1 and S2 in the Supplementary Data, for all patients and CN-AML patients, respectively. Median age was 62 years in the whole cohort and in CN-AML. CC results were available for 254 patients, of which 146 had a CN-AML. SNP-A data were available for 248 patients. Among these 248 patients, 228 were also analyzed by CC, of which 132 had a CN-AML.

### Conventional cytogenetics and SNP-A karyotyping

Among the 248 patients with available SNP-A data, we found 450 genomic aberrations in 135 patients. CNA (245 losses and 117 gains) concerned all chromosomes and UPD (n = 88) were located over all chromosomes except chromosomes 7, 14 and 18. Details of SNP-A lesions are provided in Table S3 in the Supplementary Data. Types and genomic distribution of SNP-A abnormalities were highly distinct between normal and abnormal karyotypes as defined by CC. Indeed, in AML with abnormal karyotype (n = 96), SNP-A lesions were more often CNA (222 losses and 102 gains) than UPD (n = 32). The median size was 33 Mb (range, 0.16–243) for deletions, 87 Mb (range, 0.4–249) for gains, and 24 Mb (range, 3–141) for UPDs. Most deletions were identified at chromosomes 5 (n = 28) and 7 (n = 27), gains at chromosome 8 (n = 23) and UPD at the short arm of chromosome 17 (n = 5). Nineteen patients with abnormal conventional karyotype had no detectable SNP-A lesions, including 5 with balanced chromosomal translocations or inversions, 13 with subclonal chromosomal abnormalities, and 1 with complex karyotype and several chromosome markers that may result from complex balanced translocations. In CN-AML, a total of 72 genomic aberrations were detected by SNP-A analysis in 50/132 (38%) of the patients, with a mean of 1.44 lesions per patient (range, 1–5). We detected 13 deletions (median size, 2 Mb; range, 0.15–64), 11 gains (median size, 0.5 Mb; range, 0.12–191), and 48 regions of UPDs (median size, 38 Mb; range, 2–136). Most UPDs were identified at chromosomes 13 and 1p in 7 and 6 patients, respectively (Table S3 in the Supplementary Data).

Overall, in the 228 patients analyzed by both techniques, the combination of CC and SNP-A karyotyping leads to a higher proportion of abnormal karyotypes (64%) compared with CC alone (42%) or SNP-A alone (56%) (Figure S1 in the Supplementary Data).

### Molecular findings

The incidence of gene mutations and *EVI1* overexpression in the whole cohort and in the subset of CN-AML are respectively indicated in Table 2, and Tables S1 and S2 in the Supplementary Data. At least one molecular abnormality was identified in 89% of the patients with CN-AML and 60% showed mutations in more than one of the studied genes (not shown). The description of all gene mutations identified by Sanger sequencing is provided in Table S4 in the Supplementary Data.

### Co-occurrence of genetic alterations in CN-AML

**Figure 1:**
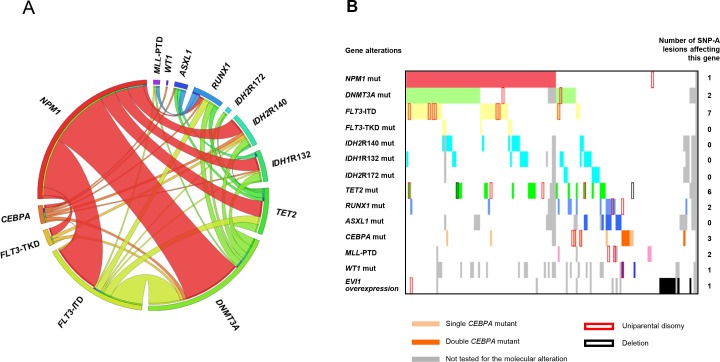
**(A)** Circos plot diagram illustrating the pairwise co-occurrence of gene mutations in cytogenetically normal acute myeloid leukemia. **(B)** Bar coding representing the co-occurrence of molecular alterations and SNP-A lesions in cytogenetically normal acute myeloid leukemia. Each patient is represented by a virtual column. Colored cells indicate the presence of a mutation in the gene(s) described in that row on the left. Abbreviations: SNP-A, single-nucleotide polymorphism array; mut, mutation; ITD, internal tandem duplication; TKD, tyrosine kinase domain; PTD, partial tandem duplication.

Integrated SNP-A karyotyping and molecular analysis in CN-AML allowed us to identify positive and negative associations between those different genetic alterations. We found that *FLT3*–ITD and *DNMT3A* mutations were significantly associated with *NPM1* mutations (P < 0.001 for both comparisons). Overall, *NPM1* mutations co-occurred with at least 1 other mutation in 90% of cases. Frequent associations between *FLT3*–ITD and *DNMT3A* mutations (P = 0.001), *RUNX1* and *ASXL1* mutations (P = 0.033), *RUNX1* and *MLL* partial tandem duplication (*MLL*-PTD) (P = 0.049) were also observed. In contrast, *RUNX1* (P = 0.009) and *CEBPA* (P = 0.033) mutations rarely co-existed with *NPM1* mutations. *NPM1* and *ASXL1* mutations were mutually exclusive (P = 0.003) (Figure 1A and 1B). *EVI1* overexpression was not found concomitantly with any gene mutation (P < 0.001) (Figure 1B).

When correlating mutational profile with SNP-A data, we found a higher incidence of *RUNX1* mutations (P = 0.01) and a lower incidence of *IDH2*R140 mutations (P = 0.03) in patients with SNP-A lesions compared to patients without SNP-A lesions (Table S5 in the Supplementary Data). We also observed that mutations affecting *CEBPA, RUNX1, DNMT3A, TET2* or *WT1* were found in 8/14 patients with UPDs (n = 12) or mono-allelic losses (n = 2) encompassing the corresponding gene locus. In addition, all 7 patients with UPD(13q) harbored a *FLT3*–ITD (Figure 1).

### Response to induction therapy

Overall, complete remission (CR) or CRp were achieved in 217 patients (202 CR and 15 CRp), including 104 patients in the control arm and 113 patients in the GO arm. In the whole cohort, the presence of unfavorable karyotype (P < 0.001), SNP-A lesion(s) (P = 0.014) were associated with a lower CR/CRp rate, in contrast to *FLT3*–ITD and *NPM1* mutations, which conferred a higher CR/CRp rate (P = 0.035 and P = 0.022, respectively). However, neither SNP-A lesion(s) nor gene mutations had an impact on response to induction therapy in the subset of CN-AML patients (Table S6 in the Supplementary Data). In multivariate analysis of the whole cohort, unfavorable karyotype remained the only factor significantly associated with a lower probability of achieving CR/CRp (P < 0.001). Randomization in the GO arm was associated with a trend (P = 0.053) towards a higher probability of CR/CRp achievement (Table S7 in the Supplementary Data).

### Prognostic analyses for relapse risk and survival

The median duration of follow-up from time to randomization was 25 months (interquartile range, 16–33). In the whole cohort, the presence of unfavorable karyotype and SNP-A lesions were predictive of a higher cumulative incidence of relapse (CIR) (P < 0.001 and P = 0.017, respectively). None of the molecular markers studied significantly influenced CIR. In the subset of CN-AML, *MLL*-PTD (P = 0.028) and *DNMT3A* mutations (P = 0.004) were both associated with an increased risk of relapse (Table 1).

**Table 1:** Incidence of genetic abnormalities and univariate prognostic analysis for cumulative incidence of relapse

	All patients				CN-AML patients			
Covariate	n (%)	HR	IC95%	P-value	n (%)	HR	IC95%	P-value
**Karyotype**								
Favorable	8/217 (3.7)	0.74	0.27–2.02	0.56	NA	NA	NA	NA
Intermediate	161/217 (74.2)	0.76	0.49–1.16	0.20	NA	NA	NA	NA
Normal karyotype	131/217 (60.4)	0.79	0.53–1.17	0.24	NA	NA	NA	NA
Unfavorable	30/217 (13.8)	2.46	1.50–4.03	**<0.001**	NA	NA	NA	NA
Not available	18/217 (8.3)	0.58	0.25–1.32	0.20	NA	NA	NA	NA
**SNP-array karyotyping**								
Presence of SNP-A lesion(s)	97/193 (50.3)	1.70	1.10–2.63	**0.017**	43/118 (36.4)	1.46	0.82–2.61	0.20
**Molecular findings**								
*MLL*-PTD	4/210 (1.9)	2.40	0.58–9.85	0.23	3/127 (2.4)	5.11	1.20–21.86	**0.028**
*NPM1* mutation	81/216 (37.5)	1.00	0.67–1.51	0.99	68/130 (52.3)	1.37	0.80–2.36	0.25
*FLT3*–TKD mutation	10/216 (4.6)	0.80	0.32–1.97	0.62	4/130 (3.1)	0.97	0.24–4.00	0.97
*FLT3*–ITD	44/216 (20.4)	1.37	0.84–2.25	0.21	33/130 (25.4)	1.54	0.85–2.79	0.15
*CEBPA* sm + dm	15/211 (7.1)	0.57	0.23–1.41	0.23	11/128 (8.6)	0.29	0.07–1.19	0.086
*CEBPA* dm	9/211 (4.3)	0.55	0.18–1.75	0.31	5/128 (3.9)	NC	NC	NC
*EVI1* overexpression	15/184 (8.1)	1.32	0.64–2.74	0.45	10/123 (8.1)	1.37	0.49–3.81	0.55
*RUNX1* mutation	20/208 (9.6)	1.50	0.82–2.76	0.19	11/125 (8.8)	1.70	0.72–4.02	0.22
*WT1* mutation	7/180 (3.9)	1.10	0.35–3.51	0.87	2/106 (1.9)	NC	NC	NC
*ASXL1* mutation	17/191 (8.9)	0.83	0.40–1.73	0.63	7/113 (6.2)	0.58	0.14–2.37	0.44
*DNMT3A* mutation	NA	NA	NA	NA	42/124 (33.9)	2.24	1.29–3.87	**0.004**
*TET2* mutation	NA	NA	NA	NA	15/125 (12.0)	0.80	0.34–1.87	0.60
*IDH1*R132 mutation	NA	NA	NA	NA	13/123 (10.6)	1.64	0.80–3.36	0.18
*IDH2*R140 mutation	NA	NA	NA	NA	12/123 (9.8)	0.47	0.17–1.31	0.15
*IDH2*R172 mutation	NA	NA	NA	NA	5/123 (4.1)	1.00	0.31–3.22	0.99

Abbreviations: CN-AML: cytogenetically normal acute myeloid leukemia; HR, hazard ratio; CI, confidence interval; SNP-A, single-nucleotide polymorphism array; NA, not applicable; NC, non-convergent; PTD, partial tandem duplication; ITD, internal tandem duplication; sm, single-mutation; dm, double-mutation.

**Figure 2:**
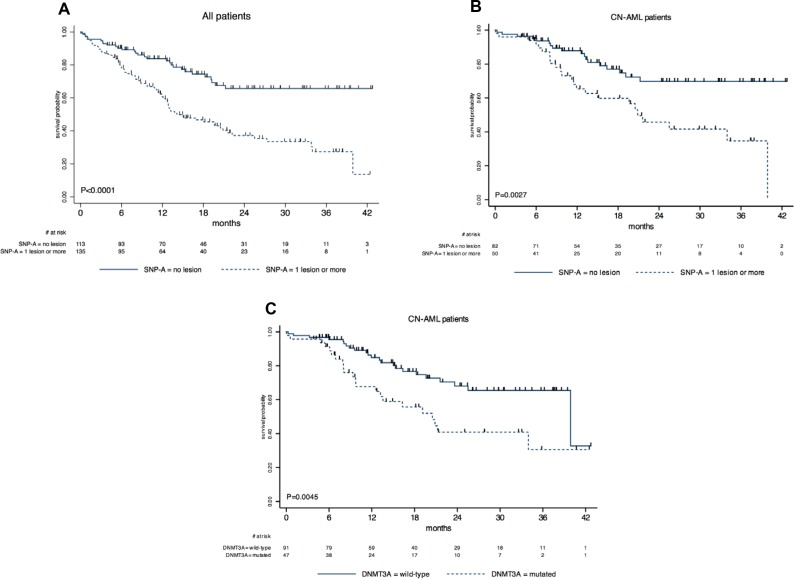
Kaplan-Meier estimates of overall survival according to SNP-array profile and gene mutational status. **(A)** in the whole patient cohort analyzed by SNP-A, 2–year OS was estimated at 68% (95%CI, 54–75) in those without SNP-A lesion *versus* 37% (95%CI, 27.5–47) in those with SNP-A lesion (P < 0.0001 by the log-rank test); **(B)** in CN-AML patients analyzed by SNP-A, 2–year OS was estimated at 70% (95%CI, 56–80) in those without SNP-A lesion *versus* 46% (95%CI, 29–61) in those with SNP-A lesion (P = 0.0027 by the log-rank test); **(C)** in CN-AML patients tested for *DNMT3A* mutation, 2–year OS was estimated at 68% (95%CI, 55–78) in those without mutation *versus* 41% (95%CI, 24–57) in those with mutation (P = 0.0045 by the log-rank test).

**Figure 3:**
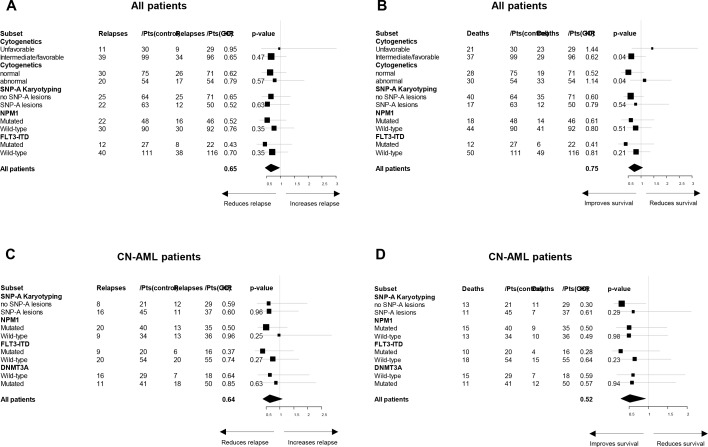
Forest plots of patient subsets and pooled data for **(A)** cumulative incidence of relapse and **(B)** overall survival in all patients, and **(C)** cumulative incidence of relapse and **(D)** overall survival in patients with cytogenetically normal acute myeloid leukemia. P-values obtained with the Gail and Simon interaction tests are also indicated.

Karyotype had a strong influence on overall survival (OS), as expected (Table 2). Interestingly, we found that OS was also significantly reduced in patients with SNP-A lesion(s), either in the whole cohort (Figure 2A) or in the subset of CN-AML (Figure 2B). In the whole cohort, *NPM1* mutations were associated with a longer OS (P = 0.022), whereas *EVI1* overexpression was associated with a shorter OS (P = 0.010). In the CN-AML subset, *DNMT3A* mutated patients had markedly reduced OS compared to *DNMT3A* wild-type patients (Figure 3C). Other molecular abnormalities studied did not significantly affect OS of CN-AML patients (Table 2).

**Table 2:** Incidence of genetic abnormalities and univariate prognostic analysis for overall survival

	All patients				CN-AML patients			
Covariate	n (%)	HR	IC95%	P-value	n (%)	HR	IC95%	P-value
**Karyotype**								
Favorable	9/278 (3.2)	0.53	0.17–1.67	0.28	NA	NA	NA	NA
Intermediate	186/278 (66.9)	0.44	0.31–0.63	**<0.001**	NA	NA	NA	NA
Normal karyotype	146/278 (52.5)	0.47	0.32–0.67	**<0.001**	NA	NA	NA	NA
Unfavorable	59/278 (21.2)	3.69	2.51–5.42	**<0.001**	NA	NA	NA	NA
Not available	24/278 (8.6)	0.76	0.37–1.57	0.47	NA	NA	NA	NA
**SNP-array karyotyping**								
Presence of SNP-A lesion(s)	135/248 (54.4)	2.62	1.71–4.03	**<0.001**	50/132 (37.9)	2.47	1.34–4.56	**0.004**
**Molecular findings**								
*MLL*-PTD	6/268 (2.2)	1.35	0.43–4.27	0.61	4/142 (2.8)	1.88	0.45–7.78	0.38
*NPM1* mutation	94/276 (34.1)	0.62	0.41–0.93	**0.022**	75/145 (51.7)	0.89	0.50–1.59	0.70
*FLT3*–TKD mutation	14/276 (5.1)	0.73	0.32–1.67	0.46	5/145 (3.4)	NC	NC	NC
*FLT3*–ITD	49/276 (17.8)	0.94	0.57–1.55	0.80	36/145 (24.8)	1.56	0.83–2.92	0.16
*CEBPA* sm + dm	18/271 (6.6)	0.60	0.26–1.37	0.23	12/143 (8.7)	0.58	0.18–1.87	0.37
*CEBPA* dm	10/271 (3.7)	0.35	0.09–1.40	0.14	5/143 (3.6)	NC	NC	NC
*EVI1* overexpression	25/251 (10.0)	2.10	1.19–3.70	**0.010**	10/135 (7.4)	1.26	0.38–4.12	0.71
*RUNX1* mutation	26/266 (9.8)	1.19	0.67–2.12	0.55	13/139 (9.4)	1.26	0.50–3.22	0.62
*WT1* mutation	7/230 (3.0)	0.64	0.16–2.61	0.54	2/119 (1.7)	NC	NC	NC
*ASXL1* mutation	21/247 (8.5)	0.87	0.44–1.73	0.70	9/127 (7.1)	1.87	0.66–5.26	0.24
*DNMT3A* mutation	NA	NA	NA	NA	47/138 (34.1)	2.29	1.27–4.11	**0.006**
*TET2* mutation	NA	NA	NA	NA	19/139 (13.7)	1.55	0.72–3.33	0.27
*IDH1*R132 mutation	NA	NA	NA	NA	15/137 (10.9)	1.18	0.53–2.66	0.69
*IDH2*R140 mutation	NA	NA	NA	NA	12/138 (8.7)	0.29	0.07–1.19	0.085
*IDH2*R172 mutation	NA	NA	NA	NA	6/137 (4.4)	0.80	0.19–3.31	0.76

Abbreviations: CN-AML: cytogenetically normal acute myeloid leukemia; HR, hazard ratio; CI, confidence interval; SNP-A, single-nucleotide polymorphism array; NA, not applicable; NC, noCN-convergent; PTD, partial tandem duplication; ITD, internal tandem duplication; sm, single-mutation; dm, double-mutation.

We then performed a multivariate analysis on CIR and OS considering the covariates previously selected as associated with clinical outcome at the 10% level, together with the treatment arm. The complete list of covariates that entered the multivariate models for CIR and OS is provided in Tables 3 and 4, respectively. The presence of unfavorable karyotype (P = 0.013) and randomization in the control arm (P = 0.007) were retained as significantly associated with higher CIR in the whole cohort, while, only the presence of *MLL*-PTD (P = 0.014) and *DNMT3A* mutations (P = 0.010) independently predicted higher CIR in CN-AML patients (Table 3). The presence of unfavorable karyotype (P<0.001) and SNP-A lesion(s) (P = 0.001) independently predicted shorter OS in the whole cohort, while the presence of SNP-A lesion(s) (P = 0.006), *DNMT3A* mutations (P = 0.042) and randomization in the control arm (P = 0.043) independently predicted shorter OS in CN-AML patients (Table 4).

**Table 3:** Multivariate analysis for cumulative incidence of relapse

	All patients			CN-AML patients		
Final model	HR	95% CI	P-value	HR	95% CI	P-value
Presence of unfavorable karyotype	2.06	1.17–3.64	**0.013**	NA	NA	NA
Presence of SNP-A lesion(s)	1.55	0.97–2.46	0.067	NA	NA	NA
Presence of *MLL*-PTD	NA	NA	NA	6.30	1.44–27.51	**0.014**
Presence of *DNMT3A* mutation	NA	NA	NA	2.12	1.20–3.75	**0.010**
Randomization in the GO arm	0.55	0.35–0.85	**0.007**	0.70	0.40–1.24	0.22

Abbreviations: HR, hazard ratio; CI, confidence interval; SNP-A, single-nucleotide polymorphism array; CN-AML: cytogenetically normal acute myeloid leukemia; NA, not applicable.

**Table 4:** Multivariate analysis for overall survival

	All patients			CN-AML patients		
Final model	HR	95% CI	P-value	HR	95% CI	P-value
Presence of unfavorable karyotype	2.54	1.56–4.12	**<0.001**	NA	NA	NA
Presence of SNP-A lesion(s)	2.25	1.39–3.65	**0.001**	2.38	1.28–4.43	**0.006**
Presence of *NPM1* mutation	1.12	0.67–1.87	0.68	NA	NA	NA
Presence of *EVI1* overexpression	1.63	0.87–3.06	0.12	NA	NA	NA
Presence of *DNMT3A* mutation	NA	NA	NA	1.91	1.02–3.56	**0.042**
Presence of *IDH2*R140 mutation	NA	NA	NA	0.38	0.09–1.63	0.19
Randomization in the GO arm	0.75	0.49–1.13	0.17	0.51	0.27–0.98	**0.043**

Abbreviations: HR, hazard ratio; CI, confidence interval; SNP–A, single–nucleotide polymorphism array; CN-AML: cytogenetically normal acute myeloid leukemia; NA, not applicable.

### Interactions between treatment effect and baseline patient characteristics

Interactions between the treatment effect on CR/CRp achievement, CIR and OS, respectively, and the following baseline patient characteristics were studied: cytogenetics, SNP-A karyotyping, *FLT3*–ITD, and *NPM1* mutations in the whole cohort and in the CN-AML subset, additionally with *DNMT3A* mutations in the CN-AML subset. When testing these potential interactions by the Gail and Simon statistics, no evidence of any statistically significant interaction was found regarding CR/CRp achievement (data not shown), CIR (Figure 3), and the variables SNP-A lesions, *DNMT3A* mutations, and *NPM1* mutations, irrespective of the endpoint.

Interestingly, we observed that the survival benefit associated with GO addition was not apparent in patients with unfavorable cytogenetics, conversely to those with favorable/intermediate cytogenetics (HR, 1.44 *versus* 0.62; test for interaction, P = 0.04; Figure 3). More specifically, CN-AML patients benefited preferentially more from GO treatment as compared to AML patients with abnormal CC (HR, 0.52 *versus* 1.14; test for interaction, P = 0.04; Figure 3).

**Figure 4:**
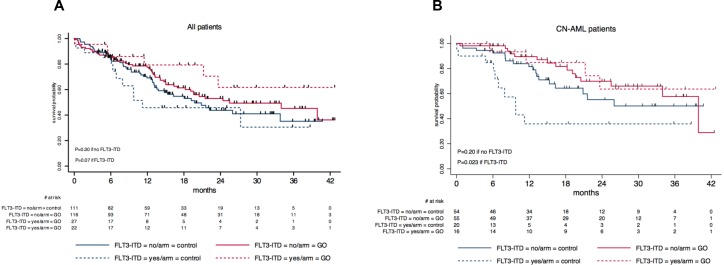
Kaplan-Meier estimates of overall survival according to *FLT3*–ITD status and treatment arm. **(A)** Overall survival according to *FLT3*–ITD status and treatment arm in all patients. In *FLT3*–ITD positive patients, 2–year OS was estimated at 46% (95%CI, 23–66) in the control arm *versus* 62% (95%CI, 32–82) in the GO arm (P = 0.07 by the log-rank test); In *FLT3*–ITD negative patients, 2–year OS was estimated at 44% (95%CI, 32–55) in the control arm *versus* 53% (95%CI, 42–63) in the GO arm (P = 0.30 by the log-rank test). **(B)** Overall survival according to *FLT3*–ITD status and treatment arm in patients with cytogenetically normal acute myeloid leukemia. In *FLT3*–ITD positive patients, 2–year OS was estimated at 36% (95%CI, 14–59) in the control arm *versus* 64% (95%CI, 29–85) in the GO arm (P = 0.023 by the log-rank test); in *FLT3*–ITD negative patients, 2–year OS was estimated at 55% (95%CI, 36–71) in the control arm *versus* 70% (95%CI, 53–82) in the GO arm (P = 0.20 by the log-rank test). Abbreviations: CN-AML: cytogenetically normal acute myeloid leukemia; ITD, internal tandem duplication.

Moreover, the OS benefit associated with GO treatment appeared more pronounced in *FLT3*–ITD positive than in negative patients, in the whole cohort as well as in CN-AML (Figure 4), although the Gail and Simon test did not reach statistical significance (Figure 3). *NPM1* mutated/*FLT3–ITD* negative genotype is considered in the ELN prognostic classification as a strong prognostic factor for favorable outcome in CN-AML [1]. In the ALFA-0701 trial, the *NPM1* mutated/*FLT3*–ITD negative genotype showed no significant influence on outcome in CN-AML (data not shown). However, when comparing the outcome of CN-AML patients with *NPM1* mutated/*FLT3*–ITD negative genotype to those with other genotypes, and according to the randomization arm, we observed that the *NPM1* mutated/*FLT3*–ITD negative genotype was associated with favorable outcome in the control arm (P = 0.04), but not in the GO arm (P = 0.89) (Figure S4).

## DISCUSSION

Our study is the first to prospectively analyze SNP-A lesions along with a large panel of gene mutations in a homogeneous cohort of adult patients with primary AML. This combined analysis allowed us to evaluate the relationships between these genetic alterations and their prognostic significance in the context of treatment with GO. Furthermore, we studied the interactions between treatment with GO and the most frequent genetic markers in AML.

Several studies in AML [18–20] have shown that SNP-A analysis allows a higher detection rate of chromosomal defects than CC, mostly due to its ability to detect small CNA and UPDs. In our cohort, we showed that, whilst having a normal conventional karyotype, 38% of the patients had ≥ 1 SNP-A lesion, UPDs being the most common abnormalities. The spectrum of gene mutations we observed is in agreement with previous studies and underlines the particularly high incidence of *NPM1* (52%), *DNMT3A* (34%), and *FLT3*–ITD (25%) mutations in older patients with CN-AML. Our study also confirms the previously reported patterns of cooperativity between gene mutations in CN-AML [11, 12, 21].

As previously described, the prognostic impact of age and white blood cell count, two well-established risk factors in AML, was not statistically significant in our cohort [13]. The only independent predictors of OS we identified in the whole cohort were unfavorable cytogenetics and SNP-A lesions. Unfavorable cytogenetics was also the only independent prognostic factor for relapse risk in the whole cohort. The potential of genetic profiling to refine prognosis in CN-AML has been highlighted by many studies [10–12, 19, 20]. In our study, multivariate analysis in CN-AML showed that presence of SNP-A lesions, *MLL*-PTD, *DNMT3A* mutations, and randomization in the control arm were associated with poor clinical outcome. Thus, our study confirmed the markedly poor prognostic impact of SNP-A lesions, both in the whole cohort and in the CN-AML subset, in accordance with previous reports [18–20]. The adverse prognostic impact of *DNMT3A* mutations in CN-AML has been consistently reported by several groups, including our own [22]. However, the molecular subgroup in which *DNMT3A* mutations are found significantly associated to a worse outcome varies across studies, most reports showing that the poor prognostic impact of *DNMT3A* mutations was most obvious in CN-AML with *NPM1*/*FLT3* non-favorable genotypes [11, 23–26]. These subgroup analyses were not relevant in the ALFA-0701 trial since the prognostic impact of *NPM1* mutated/*FLT3*–ITD negative genotype was not significant in the whole CN-AML cohort, but only in CN-AML patients treated in the control arm. In our study, the lack of favorable prognostic value of the *NPM1* mutated/*FLT3*–ITD negative genotype in CN-AML patients treated with GO can be at least partially explained by the fact that *FLT3*–ITD mutations confer a higher sensitivity to GO (see below), which is likely to compensate the benefit of being *FLT3*–ITD negative. Besides, the prognostic analysis for less common molecular alterations like *CEBPA* or *IDH1/2* mutations was limited by the small number of mutated cases in our cohort.

We and others [13–15] previously reported that the benefit of GO addition was limited to patients with cytogenetically favorable or intermediate-risk AML. Presence of *FLT3*–ITD has been recognized as a major predictor of outcome in AML, especially in the CN-AML subset [11]. Nevertheless, in the ALFA-0701 trial, *FLT3*–ITD did not significantly impact on CIR and OS in univariate analysis, suggesting that treatment with GO may improve outcome of *FLT3*–ITD positive patients. Actually, we found that the OS benefit associated with GO treatment was more pronounced in *FLT3*–ITD positive than in negative patients. Interestingly, these findings are consistent with results from Jawad *et al.* who found that leukemic stem and progenitor cells carrying *FLT3*–ITD were more sensitive to GO and that *FLT3*–ITD status was an independent predictor of *in vitro* chemosensitivity to GO [27]. However, the mechanisms underlying these differential responses remain unknown. Because GO has been withdrawn from the market, very few data on the genetic basis of response to GO in patients are available. In the MRC AML16 trial, *FLT3*–ITD mutations were found independently prognostic in older AML patients, but not predictive of response to GO. However, the cumulative dose of GO in this study was much lower than in the ALFA-0701 trial, which may account for these discordant results [15].

Overall, the current information about GO supports the efficacy of this agent in newly diagnosed AML, with acceptable toxicity [28, 29]. In light of three published randomized trials [13–15], the most appropriate indications for reapproval of GO in AML would be patients with cytogenetically favorable or intermediate-risk AML [13–15, 28, 29]. Moreover, the present data suggest that the addition of GO to standard chemotherapy may be a promising treatment option especially for *FLT3*–ITD positive AML. Although patients with *FLT3*–ITD positive AML are usually considered as candidates for allogeneic stem cell transplantation (allo-SCT), the benefit of this high-risk procedure in first CR for those patients is still a matter of debate [30–32]. During the last decade, FLT3 tyrosine kinase has emerged as an attractive therapeutic target in *FLT3* mutated AML. Several anti-FLT3 compounds are undergoing evaluation in different phases of clinical trials, as monotherapy or in combination with standard chemotherapy. Despite promising *in vitro* studies, clinical responses to single-agent FLT3 inhibitors in AML patients are generally incomplete and not sustained, and no significant improvement in OS has been demonstrated so far [33, 34]. Therefore, in *FLT3*–ITD positive AML patients, particularly in those not eligible for allo-SCT, the addition of GO to standard chemotherapy appears as an interesting treatment option, that would be worthy of further investigation in clinical trials.

In conclusion, our study emphasizes that SNP-A lesions and *DNMT3A* mutations represent adverse prognostic factors, particularly in CN-AML. Both markers add independent prognostic information and, therefore, could contribute to improve risk stratification in patients with CN-AML. Importantly, our results suggest that addition of GO to standard chemotherapy might overcome the poor prognosis of *FLT3*–ITD. However, further studies based on larger patient cohorts are required to formally identify the molecular determinants of response to GO.

## PATIENTS AND METHODS

### Patients and treatment

From January 2008 to November 2010, 278 patients aged 50–70 years with previously untreated primary AML were included in the randomized multicentric Phase 3 ALFA-0701 trial (NCT00927498), investigating the benefit of the addition of low fractionated doses of GO to standard front-line chemotherapy (Supplementary Data) [13]. The study was approved by the ethics committee of Saint-Germain en Laye, France, and by the institutional review board (IRB) of the French Regulatory Agency and undertaken in accordance with the Declaration of Helsinki. All patients provided written informed consent. Bone marrow or peripheral blood samples collected at AML diagnosis and after treatment were obtained from the tissue bank “Tumorothèque du Centre de Référence Régional en Cancérologie de Lille (C.R.R.C.)" and an approval of this study was obtained from the IRB of CHRU of Lille (CSTMT089).

### Conventional cytogenetic analysis

Cytogenetic R-banding analysis was performed on diagnostic bone marrow samples using standard methods. The karyotypes were described according to the International System for Human Cytogenetic Nomenclature recommendations [35] and classified according to the revised MRC criteria within 3 groups (favorable, intermediate and unfavorable) [36].

### Cell isolation, nucleic acid extraction, and cDNA synthesis

Mononuclear cells from pretreatment bone marrow or peripheral blood were isolated by Ficoll density gradient centrifugation (MSL, Eurobio, Courtaboeuf, France). The blast percentage following enrichment was above 60% in all samples. Genomic DNA was extracted from mononuclear cells using the QIAamp DNA Mini Kit® (Qiagen, Courtaboeuf, France) following the manufacturer’s instructions. Total RNA was extracted from the same specimen and reverse transcribed using the High-Capacity cDNA Archive Kit (Applied Biosystems, Courtaboeuf, France) and according to the standardized protocol developed within the Europe Against Cancer (EAC) program [37].

### Molecular analysis

*MLL*-PTD [38], *FLT3*–ITD [22], mutations of *FLT3* tyrosine kinase domain (FLT3–TKD) (FLT3D835/I836) [22], *NPM1* (exon 12) [39], *CEBPA* [40], *WT1* (exons 7 and 9) [41], *IDH1*R132 [42], *IDH2*R140 [43], *IDH2*R172 [42], *RUNX1* (exons 3–8) [44], *ASXL1* (exon 12) [3], *TET2* (exons 3–11) [4], and *DNMT3A* (exons 8–9, 11–23) [22] and *EVI1* overexpression [45] were assessed centrally as previously described. The screening for *TET2*, *IDH1/2*, and *DNMT3A* mutations was restricted to CN-AML. Further details regarding gene mutation analysis are available in the Supplementary Data.

### SNP-A analysis

Patient genomic DNA was processed and hybridized to Genome-Wide Human SNP 6.0 arrays (Affymetrix, Santa Clara, CA) according to the manufacturer instructions. Raw data were analyzed using the Genotyping Console version 4.1 software (Affymetrix). In order to distinguish somatic from constitutional SNP array lesions, we adopted a stringent and conservative algorithm. Aberrations were excluded as known copy number variants if there was > 50% overlap with variants from the public Database of Genomic Variants (DGV Beta version 10). Interstitial UPD < 20 Mb were considered as constitutional and excluded from the analysis. Subsequently, all CNA and UPD were validated by visual inspection and annotated based on the hg19 human genome assembly.

### Statistical analysis

Median with interquartile range and percentages were computed as summary statistics. Clinical endpoints considered for prognostic analyses were CR/CRp rate, CIR and OS. CIR and OS were calculated after censoring patients who received allo-SCT in first CR at allo-SCT time. We focused here on the prognostic influence of CC, SNP-A, and molecular alterations. First, univariate analyses for CR/CRp were based on logistic regression models with prognostic influence measured by odds ratio (OR) with 95% confidence interval (95%CI). Univariate predictive analyses for CIR and OS were based on Cox models with prognostic influence measured by hazards ratio (HR) with 95% CI. Estimation of survival curves used the Kaplan-Meier method, compared by the log-rank test [46, 47]. Stepwise multivariable Cox models allowed assessing the additional prognostic influence of those variables previously selected at the 10% level [48]. Secondly, prognostic analyses were limited to the subset of patients with CN-AML. Finally, to identify optimal treatment groups, interactions were tested using the Gail and Simon statistics [49]. This consisted of testing the heterogeneity in the OR of CR/CRp or in the HR of relapse or death according to cytogenetics, SNP-A karyotyping, and molecular findings in the two randomized treatment arms. Statistical analysis was performed with SAS (version 9.2, SAS Inc., Cary, NC) and R (version 2.13.1) softwares. All reported *P*-values are two-sided, with a nominal type I error of 0.05.

## SUPPLEMENTARY FIGURES, TABLES AND REFERENCES


