# Supplementary material for: Clinical impact of gene mutations and lesions detected by SNP-array karyotyping in acute myeloid leukemia patients in the context of gemtuzumab ozogamicin treatment: Results of the ALFA-0701 trial
Source: Oncotarget. 2014 Jan 20;5(4):916–32. doi: 10.18632/oncotarget.1536 (PMC4011594; doi:10.18632/oncotarget.1536)
Supplement: Supplementary file 1 [file oncotarget-05-916-s001.pdf]

SUPPLEMENTARY DATA

Supplement to: A Renneville, R Ben Abdelali, et *al*. Clinical impact of gene mutations and lesions detected by SNP-array karyotyping in acute myeloid leukemia patients in the context of gemtuzumab ozogamicin treatment: Results of the ALFA-0701 trial.

SUPPLEMENTARY DATA CONTENTS:

Supplementary Methods

Supplementary Tables

Table S1. Baseline patient characteristics in the whole cohort
Table S2. Baseline patient characteristics in cytogenetically normal AML
Table S3. Details of SNP-array karyotyping abnormalities
Table S4. Description of gene mutations identified by Sanger sequencing
Table S5. Comparison of molecular findings in cytogenetically normal AML according to the presence or absence of SNP array lesion
Table S6. Univariate prognostic analysis for complete remission rate (CR/CRp)
Table S7. Multivariate prognostic analysis for complete remission rate (CR/CRp) in the whole patient cohort

Supplementary Figures

Figure S1. Pie chart representing the proportion of patients with genomic abnormalities detected by conventional cytogenetics and/or SNP array karyotyping
Figure S2. Kaplan-Meier estimates of overall survival according to cytogenetics (favorable or intermediate *versus* unfavorable karyotype) and treatment arm
Figure S3. Kaplan-Meier estimates of overall survival according to cytogenetics (normal karyotype *versus* abnormal karyotype) and treatment arm
Figure S4. Kaplan-Meier estimates of overall survival according to *NPM1*/*FLT3*–ITD genotypes in cytogenetically normal AML patients

Supplementary references

SUPPLEMENTARY METHODS

Study design and patients

A prospective randomized open label phase 3 study was conducted between January 2008 and November 2010 in 26 hematological centers in France. Previously untreated patients, aged 50 to 70 years old, with a locally confirmed morphological diagnosis of acute myeloid leukemia (AML) were eligible if they had a normal cardiac function. Expression of the CD33 antigen on AML blasts was not required for study entry. Patients with prior myeloproliferative or myelodysplastic syndrome, or prior exposure to chemotherapy or radiotherapy, were not eligible. Central nervous system AML involvement, severe uncontrolled infection, as well as liver (serum transaminase level ≥ 2.5 UPN, serum bilirubin ≥ 2 UPN), or renal (serum creatinine level ≥ 2.5 UPN) dysfunction, were non-inclusion criteria. Immunophenotyping and cytogenetic analysis were performed locally. Cytogenetics was centrally collected and classified according to standard ISCN criteria[1] within three groups (favorable, intermediate, and unfavorable). Screening for gene mutations and *EVI1* overexpression and SNP array (SNP-A) karyotyping were performed centrally.

Randomization procedures

After informed consent was obtained from a patient, the investigator faxed the required registration form to the Department of Biostatistics, Saint-Louis Hospital, Paris, France. The study statistician computer-generated the random allocation sequence by use of R software (version 2.10.1). Randomization was undertaken centrally by use of telephone. Patients were stratified by center and in a one-to-one allocation ratio with block sizes of four to the control and gemtuzumab ozogamicin (GO) groups.The study was open label.

Treatment procedures

Patients received a 3+7 induction course combining intravenous daunorubicin at a dose of 60 mg/m2 on days 1 to 3 and intravenous cytarabine at a dose of 200 mg/m^2 administered by continuous infusion for 7 days without (control arm) or with GO (GO arm) at a dose of 3 mg/m^2 (maximum dose, 5 mg) over intravenous 2–hour courses on day 1, 4 and 7 after premedication with methylprednisolone. A bone marrow aspiration was performed on day 15. In case of more than 10% persistent

leukemic blasts, a second induction course was given with intravenous daunorubicin at a dose of 60 mg/m^2 for 2 days and cytarabine at a dose of 1,000 mg/m^2/12h administered intravenously over 2–hour courses for three days without additional dose of GO, followed by daily granulocyte colony-stimulating factor (lenograstim, 263 μg) until neutrophil recovery. An assessment of clinical and hematological response was done after induction therapy. Responses were classified as: complete remission (CR), defined as less than 5% blasts in a normocellular marrow and an absolute neutrophil count (ANC) of more than 1,000/μL with a platelet count of 100,000/μL or more in the peripheral blood; and CR with incomplete platelet recovery (CRp), defined as CR with residual thrombocytopenia (less than 100,000/μL). Disease progression was assessed by the study investigators and classified according to IWG criteria.[2] Patients failing to respond to induction discontinued study treatment and received a different treatment at the discretion of their treating physician. Patients in CR/CRp received two consolidation courses combining intravenous daunorubicin at a dose of 60 mg/m^2 on day 1 (first course) or for 2 days (second course) and cytarabine at a dose of 1,000 mg/m^2/12h administered intravenously over 2–hour courses of 2 hours on days 1 to 4, with or without intravenous GO at a dose of 3 mg/m^2 on day 1, according to the initial randomization. The protocol was amended in December 2009 to recommend not using GO during consolidation in patients with a platelet count less than 100,000/L by day 45 following the initiation of chemotherapy. According to the protocol, patients with non-favorable AML could be offered allogeneic stem cell transplant (SCT) if they had a matched related or unrelated donor. Assessment of clinical and hematological response was repeated before the beginning of second consolidation course, and every 3 months for 2 years. Adverse events and serious adverse events were documented according to the Common Terminology Criteria for Adverse Events (version 3.0).

Gene mutation analysis

The screening for mutations in *CEBPA*, *WT1*, *IDH1/2*, *RUNX1*, *TET2*, *ASXL1*, and *DNMT3A* was performed on genomic DNA by PCR amplification and direct Sanger sequencing. Purified PCR products were sequenced in both directions using the BigDye Terminator Cycle Sequencing Kit (Applied Biosystems) and analyzed on the Applied Biosystems 3730 Genetic Analyzer. The SeqScape software version 2.5 (Applied Biosystems) was used to detect sequence variations. Gene variations were numbered according to Genbank nucleotide sequence database. Previously annotated single nucleotide polymorphisms (SNP) (http//www.hapmap.org) were not considered pathogenic. *TET2* nonsense, frameshift, or missense variations affecting conserved domains were considered as mutations, according to Delhommeau et *al*.[3] One homozygous missense variation outside the conserved domains of TET2 (c.2570A>G; p.N857S) was still considered as a mutation because it was absent in a matched remission sample, indicating that this mutation was somatically acquired. *ASXL1* nonsense or frameshift (but not missense) variations, including the common c.1934dupG; p.G646WfsX12 variant, were considered as mutations. The complete list and description of all mutations identified by Sanger sequencing is provided in Table S4.

SUPPLEMENTARY TABLES

Table S1: Baseline patient characteristics in the whole cohort

			Control arm		GO arm		Whole cohort
**Number of patients**		**139**		**139**		**278**	
Age (years)	Median [IQR]	139	62 [57;66]	139	63 [59;67]	278	62 [58;66]
	>60	86	62%	100	72%	186	67%
	>65	41	30%	52	37%	93	33%
Male gender		61	44%	77	55%	138	50%
ECOG performance status	0	54	39%	50	36%	104	38%
	1	65	47%	75	54%	140	51%
	2	17	12%	13	9%	30	11%
	3	1	1%	1	1%	2	1%
	Not available	2		0		2	
WBC count (10^9 /L)	Median [IQR]	138	5 [1.9;26.7]	139	6.9 [2.3;30.4]	277	5.9 [2.1;29.1]
	<10	80	58%	79	57%	159	57%
	10–50	42	30%	38	27%	80	29%
	50–100	5	4%	14	10%	19	7%
	>100	11	8%	8	6%	19	7%
	Not available	1		0		1	
Platelet count (10^9 /L)	Median [IQR]	138	67.5 [36.2;125.5]	139	66 [36.5;118.5]	277	67 [36;122]
Percentage of CD33 expressing blasts	Median [IQR]	129	88 [57;96]	125	92 [67;97]	254	90 [63;97]
	>30	110	85%	112	90%	222	87%
	>90	61	47%	68	54%	129	51%
	Not available	10		14		24	
Cytogenetic risk group	Unfavorable	30	22%	29	21%	59	21%
	Favorable	6	4%	3	2%	9	3%
	Intermediate	93	67%	93	67%	186	67%
	Irrelevant*	10	7%	14	10%	24	9%
Normal karyotype		75	58%	71	57%	146	57%
SNP array karyotyping	No lesion	63	50%	50	41%	113	46%
	≥ 1 lesion	64	50%	71	59%	135	54%
	Not available	12		18		30	
Number of SNP array lesions	0	63	50%	50	41%	113	46%

(*Continued*)

		Control arm		GO arm		Whole cohort	
**Number of patients**		**139**		**139**		**278**	
	1	28	22%	34	28%	62	25%
	≥ 2	36	28%	37	31%	73	29%
	Not available	12		18		30	
NPM1	Mutated	48	35%	46	33%	94	34%
	Wild-type	90	65%	92	67%	182	66%
	Not available	1		1		2	
FLT3–ITD	Positive	27	20%	22	16%	49	18%
	Negative	111	80%	116	84%	227	82%
	Not available	1		1		2	
FLT3–TKD	Mutated	4	3%	10	7%	14	5%
	Wild-type	134	97%	128	93%	262	95%
	Not available	1		1		2	
CEBPA	Mutated	8	6%	10	7%	18	7%
	Wild-type	129	94%	124	93%	253	93%
	Not available	2		5		7	
CEBPA double mutation	Present	5	4%	5	4%	10	4%
	Absent	132	96%	129	96%	261	96%
	Not available	2		5		7	
RUNX1	Mutated	9	7%	17	13%	26	10%
	Wild-type	125	93%	115	87%	240	90%
	Not available	5		7		12	
ASXL1	Mutated	8	6%	13	11%	21	9%
	Wild-type	118	94%	108	89%	226	91%
	Not available	13		18		31	
WT1	Mutated	3	3%	4	4%	7	3%
	Wild-type	113	97%	110	96%	223	97%
	Not available	23		25		48	
MLL-PTD	Positive	3	2%	3	2%	6	2%
	Negative	133	98%	129	98%	262	98%
	Not available	3		7		10	
EVI1 overexpression	Present	12	9%	13	10%	25	10%
	Absent	115	91%	111	90%	226	90%
	Not available	12		15		27	

*The "irrelevant cytogenetic risk group" includes conventional cytogenetics not done and cytogenetics failure defined by an insufficient number of metaphases for karyotyping (less than 20 metaphases)

Table S2: Baseline patient characteristics in cytogenetically normal AML

			Control arm		GO arm		Whole cohort
**Number of patients**		**75**		**71**		**146**	
Age (years)	Median [IQR]	75	62 [57;65]	71	61 [58;65]	146	62 [58;65]
	>60	47	63%	44	62%	91	62%
	>65	22	29%	22	31%	44	30%
Male gender		34	45%	40	56%	74	51%
ECOG performance status	0	26	35%	30	42%	56	39%
	1	40	54%	34	48%	74	51%
	2	7	9%	7	10%	14	10%
	3	1	1%	0	0%	1	1%
	Not available	1		0		1	
WBC count (x 10^9 /L)	Median [IQR]	74	8.1 [2.2;29.6]	71	8.5 [2.1;30.6]	145	8.5 [2.1;30.5]
	<10	38	51%	37	52%	75	52%
	10–50	25	34%	22	31%	47	32%
	50–100	4	5%	6	8%	10	7%
	>100	7	9%	6	8%	13	9%
	Not available	1		0		1	
Platelet count (x 10^9 /L)	Median [IQR]	74	79.5 [40.5;132.8]	71	87 [48.5;136]	145	80 [46;135]
Percentage of CD33 expressing blasts	Median [IQR]	66	89.5 % [66;97]	66	93.5 % [66.5;98]	132	92 % [65;97]
	>30%	57	86%	59	89%	116	88%
	>90%	33	50%	42	64%	75	57%
	Not available	9		5		14	
SNP array karyotyping	No lesion	45	68%	37	56%	82	62%
	≥ 1 lesion	21	32%	29	44%	50	38%
	Not available	9		5		14	
Number of SNP array lesions	0	45	68%	37	56%	82	62%
	1	16	24%	20	30%	36	27%
	≥ 2	5	8%	9	14%	14	11%
	Not available	9		5		14	
NPM1	Mutated	40	54%	35	49%	75	52%
	Wild-type	34	46%	36	51%	70	48%
	Not available	1		0		1	
FLT3–ITD	Positive	20	27%	16	23%	36	25%

(*Continued*)

		Control arm		GO arm		Whole cohort	
**Number of patients**		**75**		**71**		**146**	
	Negative	54	73%	55	77%	109	75%
	Not available	1		0		1	
FLT3–TKD	Mutated	2	3%	3	4%	5	3%
	Wild-type	72	97%	68	96%	140	97%
	Not available	1		0		1	
CEBPA	Mutated	6	8%	6	9%	12	8%
	Wild-type	67	92%	64	91%	131	92%
	Not available	2		1		3	
CEBPA double mutation	Present	3	4%	2	3%	5	3%
	Absent	70	96%	68	97%	138	97%
	Not available	2		1		3	
RUNX1	Mutated	3	4%	10	14%	13	9%
	Wild-type	67	96%	59	86%	126	91%
	Not available	5		2		7	
ASXL1	Mutated	4	6%	5	8%	9	7%
	Wild-type	60	94%	58	92%	118	93%
	Not available	11		8		19	
WT1	Mutated	0	0%	2	3%	2	2%
	Wild-type	59	100%	58	97%	117	98%
	Not available	16		11		27	
MLL-PTD	Positive	2	3%	2	3%	4	3%
	Negative	71	97%	67	97%	138	97%
	Not available	2		2		4	
EVI1 overexpression	Present	3	4%	7	10%	10	7%
	Absent	65	96%	60	90%	125	93%
	Not available	7		4		11	
DNMT3A	Mutated	29	41%	18	26%	47	34%
	Wild-type	41	59%	50	74%	91	66%
	Not available	5		3		8	
TET2	Mutated	7	10%	12	18%	19	14%
	Wild-type	64	90%	56	82%	120	86%
	Not available	4		3		7	
*IDH1*R132	Mutated	8	12%	7	10%	15	11%
	Wild-type	61	88%	61	90%	122	89%
	Not available	6		3		9	

(*Continued*)

		Control arm		GO arm		Whole cohort	
**Number of patients**		**75**		**71**		**146**	
*IDH2*R140	Mutated	5	7%	7	10%	12	9%
	Wild-type	64	93%	61	90%	125	91%
	Not available	6		3		9	
*IDH2*R172	Mutated	4	6%	2	3%	6	4%
	Wild-type	65	94%	66	97%	131	96%
	Not available	6		3		9	

Table S3: Details of SNP-array karyotyping abnormalities

UPN	Type of lesion	Chromosome	Start	End	Size (kb)	Cytoband start	Cytoband end
AML patients with abnormal karyotype							
31730	UPD	4	73862219	190921709	117059	q13.3	q35.2
31730	loss	7	1	159117386	159117	all	
31892	loss	3	63353325	77606241	14253	p14.2	p12.3
32487	UPD	3	127470734	147413101	19942	q21.3	q24
32487	loss	5	97067883	160423779	63356	q15	q34
32487	loss	7	100093400	104787336	4694	q22.1	q22.3
32487	loss	11	79042968	115417686	36375	q14.1	q23.3
32604	gain	12	22913421	31982559	9069	p12.1	p11.21
32604	gain	17	61617489	78047976	16430	q23.3	q25.3
32604	gain	8	124753869	146298143	21544	q24.13	q24.3
32604	gain	19	36931956	59095126	22163	q13.12	q13.43
32604	gain	18	1	77989256	77989	all	
32604	loss	4	53773370	114896904	61124	q12	q26
32604	loss	4	175939625	185463596	9524	q34.1	q35.1
32604	loss	5	94454440	140057079	45603	q15	q31.3
32604	loss	6	1	16801806	16802	p25.3	p22.3
32604	loss	7	45290202	154511250	109221	p13	q36.3
32604	loss	12	1	133777645	133778	all	
32604	loss	16	51929665	90080100	38150	q12.1	q24.3
32604	loss	17	1	12828861	12829	p13.3	p12
32604	loss	19	29600332	36569000	6969	q12	q13.12
32886	loss	2	3133600	7238027	4104	p25.3	p25.1
32886	loss	2	215295337	216313697	1018	q34	q35
32886	loss	2	65734748	126503793	60769	p14	q14.3
32886	gain	8	1	146298143	146298	all	
32886	UPD	17	1	10710575	10711	p13.3	p12
32886	loss	5	35062617	35565349	503	p13.2	p13.2
32886	loss	5	137337233	163470506	26133	q31.2	q34
32886	loss	6	48946550	170982522	122036	p12.3	q27
32886	loss	7	1	159117386	159117	all	
32886	loss	11	84358347	106180961	21823	q14.1	q22.3
32886	loss	14	75030350	78525721	3495	q24.3	q24.3
32886	loss	18	18541646	78015057	59473	q11.1	q23

(*Continued*)

UPN	Type of lesion	Chromosome	Start	End	Size (kb)	Cytoband start	Cytoband end
32888	gain	4	1	190921709	190922	all	
32888	gain	8	1	146298143	146298	all	
32889	gain	21	1	48074364	48074	all	
33661	gain	8	1	146298143	146298	all	
33661	gain	14	1	106914000	106914	all	
33661	loss	21	35874123	37314416	1440	q22.12	q22.12
33688	gain	8	125379392	145802456	20423	q24.13	q24.3
33688	gain	11	110336181	134944770	24609	q22.3	q25
33688	loss	5	87414103	162923254	75509	q14.3	q34
33688	loss	7	118421594	159117386	40696	q31.32	q36.3
33688	loss	17	6670214	7621464	951	p13.1	p13.1
33688	loss	17	3747659	5074290	1327	p13.2	p13.2
33854	loss	7	1	159117386	159117	all	
33974	gain	22	22805223	23202509	397	q11.22	q11.22
33974	loss	20	32140254	49586241	17446	q11.22	q13.13
34006	loss	7	1	159117386	159117	all	
34047	gain	11	1	134944770	134945	all	
34837	gain	6	126029234	170982522	44953	q22.31	q27
34837	loss	7	92432391	159119708	66687	q21.2	q36.3
35313	gain	8	1	146298143	146298	all	
35313	loss	X	133347741	133588392	241	q26.2	q26.2
35504	gain	21	36904328	41402706	4498	q22.12	q22.2
35504	gain	21	10736858	29306656	18570	p11.2	q21.3
35504	loss	21	41412641	48096945	6684	q22.2	q22.3
35504	loss	21	29306656	36897913	7591	q21.3	q22.12
35751	loss	5	100867528	135718134	34851	q21.1	q31.1
35751	gain	1	150008680	249098597	99090	q21.2	q44
35751	loss	18	61202639	77989256	16787	q21.33	q23
35751	UPD	22	1	51219006	51219	all	
35892	gain	10	1	135434551	135435	all	
35892	gain	14	1	106914000	106914	all	
36046	gain	13	1	117822980	117823	all	
36046	gain	21	36961986	40731001	3769	q22.12	q22.2
36046	gain	22	1	51219006	51219	all	
36046	loss	16	1	90287523	90288	all	
36046	loss	17	1	35071572	35072	p13.3	q12

(*Continued*)

UPN	Type of lesion	Chromosome	Start	End	Size (kb)	Cytoband start	Cytoband end
36046	loss	18	1	77989256	77989	all	
36046	loss	21	40734722	48048087	7313	q22.2	q22.3
36046	loss	21	16992119	30244877	13253	q21.1	q21.3
36046	loss	y	1	57772954	57773	all	
36253	gain	11	1	134944770	134945	all	
36344	gain	6	1	170918031	170918	all	
36344	loss	y	1	57772954	57773	all	
36690	loss	2	39781566	42764146	2983	p21	p22.1
36690	loss	3	69586486	73443534	3857	p14.1	p13
36690	loss	5	89663046	157106785	67444	q14.3	q33.3
36690	gain	21	1	48074364	48074	all	
36690	loss	7	1	159117386	159117	all	
36690	loss	13	54777781	63023073	8245	q14.3	q21.31
36690	loss	16	1	90287523	90288	all	
36690	loss	18	1	77989256	77989	all	
36754	loss	5	92230259	180857866	88628	q14.3	q35.3
36754	gain	9	2985449	6966001	3981	p24.2	p24.1
36754	gain	19	5466205	16993508	11527	p13.3	p13.11
36754	gain	21	19604365	40998110	21394	q21.1	q22.2
36754	loss	17	1	21910487	21910	all p arm	
36864	loss	2	24609452	25942740	1333	p23.3	p23.3
36864	gain	11	1	134944770	134945	all	
36924	loss	y	1	57772954	57773	all	
36932	gain	8	1	146298143	146298	all	
37212	loss	5	72620990	179682493	107062	q13.2	q35.3
37212	gain	21	36813970	47983679	11170	q22.12	q22.3
37212	loss	7	1	159117386	159117	all	
37212	loss	13	1	117822980	117823	all	
37212	loss	17	1	80920583	80921	all	
37212	loss	22	1	51219006	51219	all	
37281	loss	5	75179257	180361837	105183	q13.3	q35.3
37281	gain	1	1	249250600	249251	all	
37281	gain	21	1	48074364	48074	all	
37281	gain	22	1	51219006	51219	all	
37281	gain	10	42433611	135434551	93001	all q arm	
37281	gain	14	1	106914000	106914	all	

(*Continued*)

UPN	Type of lesion	Chromosome	Start	End	Size (kb)	Cytoband start	Cytoband end
37281	gain	11	1	134944770	134945	all	
37281	UPD	16	1	90287523	90288	all	
37281	loss	17	53961115	54124366	163	p13.3	q22
37479	loss	1	1	18305634	18306	p36.33	p36.13
37479	gain	11	75003562	134944770	59941	q13.4	q25
37570	loss	7	92500542	156603827	64103	q21.2	q36.3
37570	loss	20	31777641	49148631	17371	q11.21	q13.13
37578	gain	21	14364518	48074364	33710	all q arm	
37578	loss	12	12094053	12513364	419	p13.2	p13.2
37901	loss	3	1	198022260	198022	all	
37901	loss	5	1	180857866	180858	all	
37901	loss	11	30094935	46937785	16843	p14.1	p11.2
37901	loss	12	1	133777645	133778	all	
37901	loss	17	1	46323110	46323	p13.3	q21.32
37901	loss	20	33972052	62912463	28940	q11.22	q13.33
38288	UPD	9	1	141091382	141091	all	
38288	loss	5	102723522	158784989	56061	q21.1	q33.3
38288	gain	11	54720633	134944770	80224	all q arm	
38288	gain	8	1	146298143	146298	all	
38288	gain	11	106424557	131892862	25468	q22.3	q25
38288	loss	11	1	44679389	44679	p15.5	p11.2
38288	loss	16	51798735	90287523	38489	q12.1	q24.3
38288	loss	17	1	21910487	21910	all p arm	
38288	loss	20	55222948	55961356	738	q13.31	q13.31
38288	loss	20	35276397	50741666	15465	q11.23	q13.2
38668	loss	X	1	154913754	154914	all	
38700	loss	4	181847160	183465223	1618	q34.3	q35.1
38700	loss	5	86911826	161701023	74789	q14.3	q34
38700	loss	8	142156817	144538646	2382	q24.3	q24.3
38700	loss	12	31860923	32487750	627	p11.21	p11.21
38700	loss	12	42617900	124849843	82232	q12	q24.31
38700	loss	15	94187068	102434997	8248	q26.1	q26.3
38700	loss	17	1	80920583	80921	all	
38700	loss	21	36138801	37046727	908	q22.12	q22.12
38866	loss	3	1	198022260	198022	all	
38866	loss	5	1	180857866	180858	all	

(*Continued*)

UPN	Type of lesion	Chromosome	Start	End	Size (kb)	Cytoband start	Cytoband end
38866	loss	7	1	159117386	159117	all	
38866	loss	12	10647165	24437106	13790	p13.2	12.1
38959	loss	3	97807753	145568850	47761	q11.2	q24
38959	loss	5	87277629	156850600	69573	q14.3	q33.3
38959	loss	7	61063961	159117386	98053	all q arm	
38959	UPD	17	1	21910487	21910	all p arm	
38959	loss	11	1	40273611	40274	p15.5	p12
38959	loss	12	1	133777645	133778	all	
38960	gain	8	1	146298143	146298	all	
39005	loss	4	140218567	155130661	14912	q31.1	q31.3
39005	loss	5	103417005	174644548	71228	q21.2	q35.2
39005	loss	16	63432201	90287523	26855	q21	q24.3
39005	loss	17	29148771	30634363	1486	q11.2	q11.2
39005	loss	17	18752569	21487533	2735	p11.2	p11.2
39005	loss	21	44563325	45130991	568	q22.3	q22.3
39226	loss	5	1	180857866	180858	all	
39226	gain	1	1	249250600	249251	all	
39226	gain	21	1	48074364	48074	all	
39226	loss	11	84478859	87131514	2653	q14.1	q14.2
39226	loss	16	80580878	90226000	9645	q23.2	q24.3
39226	loss	17	1	80920583	80921	all	
39226	loss	18	1	10052042	10052	p11.32	p11.22
39226	loss	19	1	59095126	59095	all	
39266	gain	4	1	190921709	190922	all	
39266	gain	13	1	117822980	117823	all	
39266	gain	8	1	146298143	146298	all	
39294	loss	6	70984647	91027017	20042	q13	q15
39294	gain	9	1	141091382	141091	all	
39294	gain	8	1	146298143	146298	all	
39513	gain	8	1	146298143	146298	all	
39979	loss	9	69977471	102743271	32766	q21.11	q31.1
40694	loss	9	71465138	89011615	17546	q21.11	q21.33
41031	loss	7	103353741	139753081	36399	q22.1	q34
41744	loss	1	6243334	9750457	3507	p36.31	p36.22
41744	loss	2	1	243048760	243049	all	
41744	loss	7	1	159117386	159117	all	

(*Continued*)

UPN	Type of lesion	Chromosome	Start	End	Size (kb)	Cytoband start	Cytoband end
41744	loss	9	1	141091382	141091	all	
41744	gain	10	1	19765062	19765	p15.3	p12.31
41744	gain	21	1	48074364	48074	all	
41744	gain	22	1	51219006	51219	all	
41744	gain	19	1	59095126	59095	all	
41744	gain	20	1	62912463	62912	all	
41744	gain	18	1	77989256	77989	all	
41744	gain	17	1	80920583	80921	all	
41744	gain	14	1	106914000	106914	all	
41744	gain	8	1	146298143	146298	all	
41744	gain	X	1	154913754	154914	all	
41744	gain	6	1	170918031	170918	all	
41744	gain	4	1	190921709	190922	all	
41744	UPD	5	66420879	180692833	114272	q12.3	q35.3
41744	loss	11	1	134944770	134945	all	
41744	loss	12	88228357	133709815	45481	q21.32	q24.33
41744	loss	15	1	102434997	102435	all	
42342	gain	6	134272071	135454492	1182	q23.2	q23.3
42342	loss	y	1	57772954	57773	all	
42612	loss	5	1	180857866	180858	all	
42612	loss	8	1	46924210	46924	all p arm	
42612	loss	9	1	141091382	141091	all	
42612	gain	8	46847522	146298143	99451	all q arm	
42612	loss	12	1	133777645	133778	all	
42612	loss	13	1	117822980	117823	all	
42612	loss	16	1	90287523	90288	all	
42612	loss	17	1	21910487	21910	all p arm	
42612	loss	18	1	77989256	77989	all	
42612	loss	21	1	48074364	48074	all	
42612	loss	22	1	51219006	51219	all	
42612	loss	X	1	154913754	154914	all	
42963	loss	9	71006117	104555236	33549	q21.11	q31.1
42963	UPD	11	1	51538030	51538	p15.5	p11.12
43032	loss	3	1	198022260	198022	all	
43032	loss	5	1	180857866	180858	all	
43032	loss	6	61973724	79931388	17958	q11.1	q14.1

(*Continued*)

UPN	Type of lesion	Chromosome	Start	End	Size (kb)	Cytoband start	Cytoband end
43032	loss	7	1	159117386	159117	all	
43032	loss	9	98468884	98873747	405	q22.32	q22.32
43032	loss	9	90773813	92018198	1244	q22.1	q22.2
43032	loss	9	35087921	44446123	9358	p11.2	p13.3
43032	loss	10	49773669	61354615	11581	q11.22	q21.2
43032	gain	21	14364518	48074364	33710	all q arm	
43032	loss	17	1	80920583	80921	all	
43032	loss	18	1	77989256	77989	all	
43032	loss	19	1	6233345	6233	p13.3	p13.3
43032	loss	19	16310641	44500478	28190	p13.11	q13.31
43032	loss	y	1	57772954	57773	all	
43049	gain	8	1	146298143	146298	all	
43251	loss	2	24170213	25574066	1404	p23.3	p23.3
43251	loss	5	56488164	87780432	31292	q11.2	q14.3
43251	loss	5	87414103	164259724	76846	q14.3	q34
43251	loss	7	1	159117386	159117	all	
43251	loss	12	10188966	22203084	12014	p13.2	p12.1
43251	loss	13	33210841	35959600	2749	q13.1	q13.3
43251	gain	8	1	146298143	146298	all	
43251	UPD	20	6783273	9791900	3009	p12.3	p12.2
43251	UPD	6	166048867	170918031	4869	q27	q27
43251	UPD	20	55437710	62912463	7475	q13.31	q13.33
43251	UPD	X	143877892	155233846	11356	q27.3	q28
43251	UPD	12	58410755	80838012	22427	q14.1	q21.31
43251	UPD	9	88001520	113081565	25080	q21.33	q31.3
43251	UPD	15	30263991	57654706	27391	q13.1	q21.3
43251	UPD	11	69964055	111168024	41204	q13.3	q23.1
43251	UPD	X	43697637	86624994	42927	p11.3	q21.31
43251	UPD	3	7026695	61469903	54443	p26.1	p14.2
43251	loss	17	7016238	8080589	1064	p13.1	p13.1
43251	loss	X	38639370	39959415	1320	p11.4	p11.4
43455	gain	13	1	117822980	117823	all	
43803	gain	8	1	146298143	146298	all	
43803	gain	X	1	154913754	154914	all	
43803	gain	5	1	180857866	180858	all	
43803	gain	13	1	117822980	117823	all	

(*Continued*)

UPN	Type of lesion	Chromosome	Start	End	Size (kb)	Cytoband start	Cytoband end
43803	gain	10	1	135434551	135435	all	
43920	loss	6	166862319	168240549	1378	q27	q27
44014	gain	8	13353847	14667346	1313	p22	p22
44288	loss	3	1	198022260	198022	all	
44288	loss	4	101226126	142283442	41057	q24	q31.21
44288	loss	5	86654812	157080784	70426	q14.3	q33.3
44288	loss	6	71594431	114165133	42571	q13	q21
44288	loss	7	126877411	159119708	32242	q31.33	q36.3
44288	loss	12	1	45405040	45405	p13.33	q12
44288	loss	13	49570629	51615913	2045	q14.2	q14.3
44288	loss	14	91374032	91767733	394	q32.11	q32.11
44288	loss	14	95361225	96230379	869	q32.13	q32.13
44288	gain	9	132448018	141091382	8643	q34.11	q34.4
44288	gain	8	1	146298143	146298	all	
44288	UPD	17	1	9976637	9977	p13.3	p13.1
44288	loss	20	34187312	50599678	16412	q11.22	q13.2
44755	loss	7	1	5454831	5455	p22.3	p22.1
44755	loss	7	101872313	159119708	57247	q22.1	q36.3
44755	loss	20	30851171	43375745	12525	q11.21	q13.12
44757	loss	1	156780305	158979950	2200	q23.1	q23.1
44757	loss	4	53447267	55104638	1657	q12	q12
44757	loss	5	66327006	180719790	114393	q12.3	q35.3
44757	loss	13	44512930	45959089	1446	q14.11	q14.13
44757	loss	13	50168964	51686166	1517	q14.2	q14.3
44757	loss	16	29139020	90146944	61008	p11.2	q24.3
44757	gain	1	1	99597650	99598	p36.33	p21.3
44757	gain	21	33551887	48042692	14491	q22.11	q22.3
44757	loss	17	59360247	60022976	663	q23.2	q23.2
44757	loss	17	1	21910487	21910	all p arm	
44757	loss	18	1	77989256	77989	all	
44757	loss	21	15372508	25119271	9747	q11.2	q21.2
44757	loss	y	1	57772954	57773	all	
44794	gain	1	144007036	249250600	105244	all q arm	
44794	gain	19	1	24313483	24313	all p arm	
44814	gain	8	1	146298143	146298	all	
46051	UPD	5	166331864	180692833	14361	q34	q35.3

(*Continued*)

UPN	Type of lesion	Chromosome	Start	End	Size (kb)	Cytoband start	Cytoband end
46051	UPD	8	102123117	124710664	22588	q22.3	q24.13
46237	loss	4	105493834	106986267	1492	q24	q24
46237	loss	5	87677784	157946648	70269	q14.3	q33.3
46237	loss	7	1	159117386	159117	all	
46533	loss	5	126977517	180719790	53742	q23.2	q35.3
46533	loss	7	1	54108818	54109	p22.3	p11.2
46533	loss	12	11931862	14786074	2854	p13.2	p13.1
46533	UPD	12	1	30931399	30931	p13.33	p11.21
46608	gain	8	1	146298143	146298	all	
46608	UPD	11	65140208	134944770	69805	q13.1	q25
46716	UPD	10	119681400	135434551	15753	q26.11	q26.3
46953	loss	7	1	159117386	159117	all	
46953	gain	20	648252	4556395	3908	p13	p13
46953	gain	20	55861274	60090382	4229	q13.31	q13.33
46953	gain	20	18343107	31059295	12716	p11.23	q11.21
46953	UPD	11	733638	51563636	50830	p15.5	p11.12
46953	loss	20	4827190	17859779	13033	p13	p12.1
46953	loss	20	31325453	50274179	18949	q11.21	q13.2
46953	loss	21	35090967	36564197	1473	q22.11	q22.12
47173	loss	1	165608440	194476973	28869	q24.1	q31.3
47173	loss	6	99833911	105150619	5317	q16.2	q16.3
47173	loss	11	15350431	16119232	769	p15.2	p15.2
47175	loss	2	85667355	86934733	1267	p11.2	p11.2
47175	loss	5	9883139	30745775	20863	p15.2	p13.3
47175	loss	5	49455623	180361837	130906	all q arm	
47175	loss	7	101148414	159067015	57919	q22.1	q36.3
47175	loss	11	94093387	134944770	40851	q21	q25
47175	loss	13	19482796	84284509	64802	q11	q31.1
47175	loss	17	12536406	13500666	964	p12	p12
47175	loss	17	27760142	47806474	20046	q11.2	q21.33
47175	loss	18	30380422	32642676	2262	q12.1	q12.1
47175	loss	18	1	5651168	5651	p11.32	p11.31
47175	loss	18	63794727	78015057	14220	q22.1	q23
47175	loss	19	13218305	21185295	7967	p13.2	p12
47175	gain	1	1	9502485	9502	p36.33	p36.22
47175	gain	5	30781937	37717093	6935	p13.3	p13.2

(Continued)

UPN	Type of lesion	Chromosome	Start	End	Size (kb)	Cytoband start	Cytoband end
47175	gain	21	11039259	23709090	12670	p11.1	q21.1
47175	gain	21	27380444	48096945	20717	q21.3	q22.3
47175	gain	18	32645452	63753423	31108	q12.1	q22.1
47175	gain	19	27747211	59095126	31348	all q arm	
47175	UPD	17	1	12610676	12611	p13.3	p12
47175	loss	21	24683489	27380444	2697	q21.2	q21.3
47175	loss	X	1	154913754	154914	all	
47202	gain	11	1	134944770	134945	all	
47283	loss	7	1	159117386	159117	all	
47283	gain	21	38299015	40254480	1955	q22.13	q22.2
47283	UPD	6	87263957	170918031	83654	q14.3	q27
47542	loss	3	116102401	116966965	865	q13.31	q13.31
47542	loss	7	1	159117386	159117	all	
47542	gain	8	1	146298143	146298	all	
47542	UPD	17	1	3394299	3394	p13.3	p13.2
47542	UPD	20	1	5516511	5517	p13	p12.3
47542	UPD	11	66240190	134944770	68705	q13.2	q25
47809	loss	7	1	159117386	159117	all	
48418	gain	13	1	117822980	117823	all	
48418	UPD	21	30613465	48084820	17471	q21.3	q22.3
48418	loss	X	39607142	40053409	446	p11.4	p11.4
48570	loss	3	17910235	19921288	2011	p24.3	p24.3
48570	loss	3	70993294	73100009	2107	p13	p13
48570	loss	12	1	133777645	133778	all	
48570	loss	13	44478804	53059204	8580	q14.11	q14.3
48570	loss	16	88626588	90104236	1478	q24.2	q24.3
48570	gain	13	100116568	115108385	14992	q32.3	q34
48570	gain	13	19853295	42359173	22506	q12.11	q14.11
48570	loss	X	117322957	139000449	21677	q24	q27.1
48936	loss	5	106627590	150682211	44055	q21.3	q33.1
48936	loss	8	124488120	132146269	7658	q24.13	q24.22
48936	loss	15	56483603	61524603	5041	q21.3	q22.2
48936	loss	17	26872147	28037184	1165	q11.2	q11.2
48936	loss	17	563766	8282911	7719	p13.3	p13.1
49776	gain	8	1	146298143	146298	all	

(*Continued*)

UPN	Type of lesion	Chromosome	Start	End	Size (kb)	Cytoband start	Cytoband end
AML patients with normal karyotype							
31871	UPD	6	1	4602821	4603	p25.3	p25.1
31871	UPD	1	1	53762781	53763	p36.33	p32.3
31871	gain	12	84028416	84450824	422	q21.31	q21.31
32433	UPD	20	32482631	60142609	27660	q13.2	q13.33
32433	UPD	16	14008673	51320672	37312	p13.12	q12.1
33393	gain	1	2694432	3208375	514	p36.32	p36.32
33448	loss	13	46024532	52457382	6433	q14.13	q14.3
33595	loss	16	15416497	16644230	1228	p13.11	p13.11
33623	UPD	6	149523575	170918031	21394	q25.1	q27
33623	UPD	X	132300889	155233846	22933	q26.2	q28
33623	UPD	3	127247373	170244995	42998	q21.3	q26.2
33623	UPD	6	45765252	91191316	45426	p21.1	q15
34015	UPD	11	1	38400522	38401	all p arm	
34029	loss	14	65738549	65892724	154	q23.3	q23.3
34170	UPD	1	1	15153369	15153	p36.33	p36.21
34189	UPD	11	63864210	134117015	70253	q13.1	q25
34441	UPD	4	105455032	190921709	85467	q24	q35.2
34441	UPD	13	19045111	117822980	98778	all	
35425	loss	4	93137627	107307023	14169	q22.1	q24
35617	UPD	13	19045111	117822980	98778	all	
36656	gain	5	124870864	126034989	1164	q23.2	q23.2
36926	UPD	2	1	86376528	86377	p25.3	p11.2
36979	UPD	19	32824309	59095126	26271	q13.11	q13.43
37316	UPD	13	19045111	117822980	98778	all q arm	
37316	gain	19	28232667	29655286	1423	q11	q12
37364	UPD	20	60090381	62912463	2822	q13.33	q13.33
37415	UPD	13	19045111	117822980	98778	all q arm	
37480	UPD	4	79515723	190921709	111406	q21.21	q35.2
37538	UPD	5	140646420	180208930	39563	q31.3	q35.3
37736	UPD	4	101463176	190921709	89459	q24	q35.2
38121	gain	21	27753016	28593295	840	q21.3	q21.3
38286	gain	9	134482549	134697513	215	q34.13	q34.13
38373	UPD	8	1	12481979	12482	p23.3	p23.1
38373	UPD	11	113893878	132030757	18137	q23.2	q25

(*Continued*)

UPN	Type of lesion	Chromosome	Start	End	Size (kb)	Cytoband start	Cytoband end
39248	UPD	16	62465328	90163275	27698	q21	q24.3
39798	UPD	1	1	53120303	53120	p36.33	p32.3
40879	UPD	13	19045111	117822980	98778	all	
40883	UPD	1	1	121291080	121291	p36.33	p11.2
41529	UPD	8	107948824	128480984	20532	q23.1	q24.21
41529	UPD	12	111793038	133777645	21985	q24.12	q24.33
41529	UPD	16	12871638	51108237	38237	p13.12	q12.1
41529	UPD	12	3664703	76537112	72872	p13.32	q21.2
42520	UPD	19	28914912	59095126	30180	q12	q13.43
42595	loss	7	92299717	156790714	64491	q21.2	q36.3
42749	gain	4	1	190921709	190922	all	
42784	UPD	1	70226172	90928816	20703	p31.1	p22.2
43516	gain	11	62041886	62161393	120	q12.3	q12.3
43516	UPD	6	42821806	64762195	21940	p21.1	q12
43516	UPD	5	114445574	142529699	28084	q22.3	q31.3
43516	UPD	2	18287149	53847886	35561	p24.2	p16.2
43516	UPD	5	32053320	73508989	41456	p13.3	q13.3
43606	UPD	4	1	3463882	3464	p16.3	p16.3
44348	loss	12	9731636	13018781	3287	p13.31	p13.1
44978	UPD	13	19045111	117822980	98778	all q arm	
46839	loss	20	48658311	49152156	494	q13.13	q13.13
46945	loss	4	105661081	106121342	460	q24	q24
46946	loss	5	157302786	158536500	1234	q33.3	q33.3
46946	UPD	16	46534789	90055669	43521	all q arm	
48483	UPD	4	52696791	188905061	136208	q11	q35.2
48868	UPD	21	32578621	47974075	15395	q22.11	q22.3
48934	loss	9	21119163	21283847	165	p21.3	p21.3
48934	UPD	13	19045111	117822980	98778	all q arm	
48962	gain	17	28979006	29283712	305	q11.2	q11.2
49444	gain	2	122149522	122653612	504	q14.2	q14.3
49444	loss	17	29309889	31298767	1989	q11.2	q11.2
49444	UPD	21	30807998	48084820	17277	q21.3	q22.3
49650	loss	3	65207318	83907001	18700	p14.1	p12.1
49886	UPD	1	1	119424026	119424	all p arm	
50645	loss	13	103700865	106773570	3073	q33.1	q33.2
50645	UPD	22	17990257	51219006	33229	q11.21	q13.33
94	UPD	19	32829305	59095126	26266	q13.11	q13.43

(*Continued*)

UPN	Type of lesion	Chromosome	Start	End	Size (kb)	Cytoband start	Cytoband end
AML patients with irrelevant cytogenetics							
33639	UPD	12	66256946	133777645	67521	q14.3	q24.33
33730	UPD	6	1	35688619	35689	p25.3	p21.31
34392	UPD	5	138405134	180634876	42230	q31.2	q35.3
37322	UPD	2	1	63959535	63960	p25.3	p15
38772	UPD	13	19045111	117822980	98778	all	
38772	UPD	6	1	35939688	35940	p25.3	p21.31
43792	loss	2	133809378	133978870	169	q21.2	q21.2
43792	loss	12	93293984	95002431	1708	q22	q22
46534	loss	4	1	190921709	190922	all	
46534	loss	5	49455623	180361837	130906	all q arm	
46534	loss	7	1	159117386	159117	all	
46534	loss	14	34886955	46414289	11527	q13.1	q21.2
46534	loss	17	1	80920583	80921	all	
46534	gain	2	29564717	83707576	54143	p23.2	p11.2
46534	gain	7	26526959	27849414	1322	p15.2	p15.2
46534	gain	13	27577946	29940249	2362	q12.13	q12.3
46534	gain	21	14560697	30107787	15547	q11.2	q21.3
46534	UPD	2	1	29632572	29633	p25.3	p23.2
46534	UPD	2	82739795	243048760	160309	p12	q37.3
46534	loss	21	30110612	48074364	17964	q21.3	q22.3
46534	loss	X	1539702	44816937	43277	p22.33	p11.3
49413	loss	16	74695078	74869794	175	q23.1	q23.1

Abbreviations: UPN, unique patient number; UPD, uniparental disomy.
Start: first base pair location of the copy number abnormality or UPD region. End: last base pair location of the copy number abnormality or UPD region.

Table S4: Description of gene mutations identified by Sanger sequencing

Gene	Reference transcript	Exon	Type of mutation	Nucleotide variation	Protein variation	Status	Number of mutations identified
ASXL1	NM_015338.5	12	Frameshift	c.1934dupG	p.G646WfsX12	Het	12
ASXL1	NM_015338.5	12	Frameshift	c.1900_1922del	p.E635RfsX16	Het	1
ASXL1	NM_015338.5	12	Nonsense	c.2077C>T	p.R693X	Het	1
ASXL1	NM_015338.5	12	Frameshift	c.1989_1996del	p.S663RfsX2	Het	1
ASXL1	NM_015338.5	12	Frameshift	c.2505del	p.T836LfsX2	Het	1
ASXL1	NM_015338.5	12	Nonsense	c.2873C>A	p.S958X	Het	1
ASXL1	NM_015338.5	12	Nonsense	c.2324T>A	p.L775X	Het	1
CEBPA	NM_004364.2	1	Frameshift	c.69_78del	p.H24AfsX133	Het	1
CEBPA	NM_004364.2	1	Frameshift	c.95dupG	p.F33LfsX75	Het	1
CEBPA	NM_004364.2	1	Frameshift	c.103delC	p.R35GfsX125	Het	1
CEBPA	NM_004364.2	1	Frameshift	c.109_121del	p.A37SfsX119	Het	1
CEBPA	NM_004364.2	1	Nonsense	c.169G>T	p.E57X	Het	1
CEBPA	NM_004364.2	1	Frameshift	c.197_198delinsTAG	p.A66VfsX41	Het	1
CEBPA	NM_004364.2	1	Frameshift	c.198dupC	p.Y67LfsX40	Het	1
CEBPA	NM_004364.2	1	Frameshift	c.209dupC	p.A71GfsX37	Het	1
CEBPA	NM_004364.2	1	Frameshift	c.238_241dupGACC	p.L81RfsX27	Het	1
CEBPA	NM_004364.2	1	Frameshift	c.247dupC	p.Q83PfsX24	Het	1
CEBPA	NM_004364.2	1	Frameshift	c.277_280dup	p.A94GfsX15	Het	1
CEBPA	NM_004364.2	1	Frameshift	c.309delC	p.G104AfsX56	Het	1
CEBPA	NM_004364.2	1	Frameshift	c.324C>G	p.Y108X	Het	1
CEBPA	NM_004364.2	1	Frameshift	c.336_343delinsG	p.A113PfsX45	Het	1
CEBPA	NM_004364.2	1	Frameshift	c.342delC	p.G116AfsX44	Het	1
CEBPA	NM_004364.2	1	In-frame deletion	c.876_878del	p.N293del	Het	1
CEBPA	NM_004364.2	1	In-frame duplication	c.898_942dup	p.R300_V314dup	Het	1
CEBPA	NM_004364.2	1	In-frame duplication	c.904_906dup	p.K302dup	Het	1
CEBPA	NM_004364.2	1	In-frame insertion	c.911_912insGTT	p.K304_Q305insL	Het	1
CEBPA	NM_004364.2	1	In-frame insertion	c.924_925insGGA	p.V308_E309insG	Het	1
CEBPA	NM_004364.2	1	In-frame insertion	c.924delGinsAACT	p.V308_E309insT	Het	1
CEBPA	NM_004364.2	1	In-frame duplication	c.929_930insC and c.907_929dup	p.K303_T310dup	Het	1

(Continued)

Gene	Reference transcript	Exon	Type of mutation	Nucleotide variation	Protein variation	Status	Number of mutations identified
CEBPA	NM_004364.2	1	In-frame duplication	c.930_932dup	p.Q312dup	Het	1
CEBPA	NM_004364.2	1	In-frame duplication	c.933_935dup	p.K313dup	Het	1
CEBPA	NM_004364.2	1	In-frame insertion	c.940_941insAAG	p.K313_V314insE	Het	1
CEBPA	NM_004364.2	1	In-frame insertion	c.950_951ins(CA + 908_950dup)	p.L317_T318ins(T + Q304_L317dup)	Hom	1
CEBPA	NM_004364.2	1	In-frame insertion	c.970_971ins36bp	p.R323_L324ins RKVLELTSDNDR	Het	1
CEBPA	NM_004364.2	1	In-frame deletion	c.1064_1069del	p.N356_C357del	Het	1
DNMT3A	NM_022552.3	8	Missense	c.901C>T	p.R301W	Het	1
DNMT3A	NM_022552.3	8	In-frame deletion	c.904_906del	p.G302del	Het	1
DNMT3A	NM_022552.3	8	Frameshift	c.943_971del	p.M315Hfsx18	Hom	1
DNMT3A	NM_022552.3	8	Nonsense	c.958C>T	p.R320X	Het	1
DNMT3A	NM_022552.3	9	Missense	c.1055G>A	p.S352N	Het	1
DNMT3A	NM_022552.3	11	Nonsense	c.1308C>A	p.Y436X	Het	1
DNMT3A	NM_022552.3	NA	Splice site (intron 11)	c.1430–1G>A	NA	Het	1
DNMT3A	NM_022552.3	14	Missense	c.1574C>A	p.A525E	Het	1
DNMT3A	NM_022552.3	14	Frameshift	c.1605delC	p.Y536TfsX115	Het	1
DNMT3A	NM_022552.3	14	Missense	c.1627G>T	p.G543C	Het	3
DNMT3A	NM_022552.3	15	Missense	c.1711G>C	p.A571P	Het	1
DNMT3A	NM_022552.3	15	Nonsense	c.1792C>T	p.R598X	Het	2
DNMT3A	NM_022552.3	15	Nonsense	c.1816C>T	p.Q606X	Het	1
DNMT3A	NM_022552.3	16	Missense	c.1925G>T	p.G642V	Het	1
DNMT3A	NM_022552.3	18	Missense	c.2096G>A	p.G699D	Hom	1
DNMT3A	NM_022552.3	18	Missense	c.2098C>T	p.P700S	Het	1
DNMT3A	NM_022552.3	18	Missense	c.2116G>A	p.G706R	Het	1
DNMT3A	NM_022552.3	18	Missense	c.2141C>G	p.S714C	Het	5
DNMT3A	NM_022552.3	19	Missense	c.2264T>C	p.F755S	Het	1
DNMT3A	NM_022552.3	19	Missense	c.2312G>A	p.R771Q	Het	1
DNMT3A	NM_022552.3	20	Missense	c.2339T>C	p.I780T	Het	1
DNMT3A	NM_022552.3	20	Nonsense	c.2379C>A	p.Y793X	Het	1
DNMT3A	NM_022552.3	22	Frameshift	c.2552delT	p.F851SfsX2	Het	1

(*Continued*)

Gene	Reference transcript	Exon	Type of mutation	Nucleotide variation	Protein variation	Status	Number of mutations identified
DNMT3A	NM_022552.3	23	Missense	c.2624C>T	p.T875I	Het	1
DNMT3A	NM_022552.3	23	Missense	c.2644C>A	p.R882S	Het	1
DNMT3A	NM_022552.3	23	Missense	c.2644C>T	p.R882C	Het	6
DNMT3A	NM_022552.3	23	Missense	c.2645G>A	p.R882H	Het	9
DNMT3A	NM_022552.3	23	Missense	c.2645G>C	p.R882P	Het	1
DNMT3A	NM_022552.3	23	Missense	c.2656C>G	p.Q886E	Het	1
DNMT3A	NM_022552.3	23	Missense	c.2682C>G	p.S894R	Het	1
DNMT3A	NM_022552.3	23	Missense	c.2696G>A	p.R899H	Het	1
DNMT3A	NM_022552.3	23	Missense	c.2732G>A	p.C911Y	Het	1
IDH1	NM_005896.2	4	Missense	c.394C>T	p.R132C	Het	5
IDH1	NM_005896.2	4	Missense	c.395G>A	p.R132H	Het	5
IDH1	NM_005896.2	4	Missense	c.394C>G	p.R132G	Het	3
IDH1	NM_005896.2	4	Missense	c.394C>A	p.R132S	Het	2
IDH2	NM_002168.2	4	Missense	c.419G>A	p.R140Q	Het	12
IDH2	NM_002168.2	4	Missense	c.525G>A	p.R172K	Het	6
NPM1	NM_002520.6	12	Frameshift	c.860_863dupTCTG	p.W288CfsX12	Het	71
NPM1	NM_002520.6	12	Frameshift	c.863_864insCATG	p.W288CfsX12	Het	10
NPM1	NM_002520.6	12	Frameshift	c.863_864insCCTG	p.W288CfsX12	Het	7
NPM1	NM_002520.6	12	Frameshift	c.863_864insCTTG	p.W288CfsX12	Het	3
NPM1	NM_002520.6	12	Frameshift	c.863_864insTATG	p.W288CfsX12	Het	2
NPM1	NM_002520.6	12	Frameshift	c.863_864insCGTC	p.W288CfsX12	Het	1
RUNX1	NM_001001890	3	Frameshift	c.182_183dupAG	p.L62SfsX34	Het	1
RUNX1	NM_001001890	3	Frameshift	c.218_219insCCT	p.V74PfsX382	Het	1
RUNX1	NM_001001890	3	Missense	c.236G>C	p.W79S	Hom	1
RUNX1	NM_001001890	3	Missense	c.239G>A	p.R80H	Het	1
RUNX1	NM_001001890	4	Frameshift	c.342_343insCTTG	p.A115LfsX3	Het	1
RUNX1	NM_001001890	5	Missense	c.397G>A	p.D133N	Het	1
RUNX1	NM_001001890	4	Missense	c.401T>G	p.L134R	Het	1
RUNX1	NM_001001890	4	Missense	c.404G>C	p.R135K	Het	1
RUNX1	NM_001001890	4	Missense	c.413G>A	p.G138D	Het	1
RUNX1	NM_001001890	4	Missense	c.416G>A	p.R139Q	Het	1
RUNX1	NM_001001890	4	Missense	c.416G>A	p.R139Q	Hom	1
RUNX1	NM_001001890	5	Missense	c.501A>T	p.K167N	Het	1
RUNX1	NM_001001890	5	Missense	c.512A>T	p.D171V	Het	2
RUNX1	NM_001001890	5	Nonsense	c.520C>T	p.R174X	Het	1

(*Continued*)

Gene	Reference transcript	Exon	Type of mutation	Nucleotide variation	Protein variation	Status	Number of mutations identified
RUNX1	NM_001001890	5	Missense	c.521G>A	p.R174Q	Het	1
RUNX1	NM_001001890	5	Missense	c.530G>A	p.R177Q	Het	1
RUNX1	NM_001001890	6	Missense	c.538C>T	p.R180W	Het	2
RUNX1	NM_001001890	6	Nonsense	c.607C>T	p.Q203X	Het	1
RUNX1	NM_001001890	6	Frameshift	c.698_699insATTAA	p.N233KfsX53	Het	1
RUNX1	NM_001001890	6	Nonsense	c.703C>T	p.Q235X	Het	1
RUNX1	NM_001001890	7	Nonsense	c.877C>T	p.R293X	Het	4
RUNX1	NM_001001890	8	Frameshift	c.923_924insA	p.Q308QfsX265	Het	1
RUNX1	NM_001001890	8	Nonsense	c.1349G>A	p.W450X	Het	1
TET2	NM_001127208	3	Frameshift	c.483dupA	p.D162RfsX9	Het	1
TET2	NM_001127208	3	Frameshift	c.1842dupG	p.L615AfsX23	Het	1
TET2	NM_001127208	3	Nonsense	c.2053C>T	p.Q685X	Het	1
TET2	NM_001127208	3	Frameshift	c.2083_2105dup	p.H702QfsX6	Het	1
TET2	NM_001127208	3	Nonsense	c.2158C>T	p.Q720X	Het	1
TET2	NM_001127208	3	Frameshift	c.2222_2231del	p.N741SfsX7	Het	1
TET2	NM_001127208	3	Frameshift	c.2290delC	p.Q764KfsX49	Het	1
TET2	NM_001127208	3	Frameshift	c.2348_2367del	p.E783AfsX12	Hom	1
TET2	NM_001127208	3	Nonsense	c.2428C>T	p.Q810X	Het	1
TET2	NM_001127208	3	Nonsense	c.2474delC	p.S825X	Het	1
TET2	NM_001127208	3	Frameshift	c.2565delA	p.A855AfsX18	Het	1
TET2	NM_001127208	3	Missense	c.2570A>G	p.N857S	Hom	1
TET2	NM_001127208	3	Nonsense	c.2746C>T	p.Q916X	Het	1
TET2	NM_001127208	6	Nonsense	c.3646C>T	p.R1216X	Het	1
TET2	NM_001127208	6	Missense	c.3671C>T	p.A1224V	Het	1
TET2	NM_001127208	7	Frameshift	c.3812dupG	p.C1271WfsX29	Het	1
TET2	NM_001127208	7	Missense	c.3860T>C	p.F1287S	Het	1
TET2	NM_001127208	8	Missense	c.4042C>A	p.Q1348K	Het	1
TET2	NM_001127208	9	Missense	c.4102T>C	p.F1368L	Het	1
TET2	NM_001127208	9	Missense	c.4138C>T	p.H1380Y	Het	1
TET2	NM_001127208	9	Missense	c.4178C>T	p.T1393I	Het	1
TET2	NM_001127208	10	Missense	c.4255C>T	p.P1419S	Het	1
TET2	NM_001127208	10	Nonsense	c.4354C>T	p.R1452X	Het	1
TET2	NM_001127208	11	Frameshift	c.5102delT	p.M1701SfsX18	Het	1
WT1	NM_024426	7	Frameshift	c.923_924insCC	p.T309RfsX73	Het	1
WT1	NM_024426	7	Frameshift	c.929_938 dup	p.A314CfsX6	Het	1

(*Continued*)

Gene	Reference transcript	Exon	Type of mutation	Nucleotide variation	Protein variation	Status	Number of mutations identified
WT1	NM_024426	7	Frameshift	c.933_934ins AGGACATCCC	p.S313TfsX7	Het	1
WT1	NM_024426	7	Frameshift	c.937_940dup	p.A314VfsX4	Het	1
WT1	NM_024426	7	Frameshift	c.938_939insCGGTC	p.A314GfsX69	Het	1
WT1	NM_024426	7	Frameshift	c.944_945insCG	p.E316VfsX66	Het	1
WT1	NM_024426	9	Missense	c.1180C>G	p.R394G	Het	1

Abbreviations: Het, heterozygous; Hom, homozygous.

Table S5: Comparison of molecular findings in cytogenetically normal AML according to the presence or absence of SNP array lesion

	No SNP-array lesion		≥ 1 SNP-array lesion(s)		P-value
	N	%	N	%	
**Number of patients**	**82**		**50**		
**Molecular findings**					
MLL-PTD	1	1.22 %	3	6.12 %	0.15
NPM1 mutation	48	58.54 %	23	46%	0.21
FLT3–TKD mutation	5	6.1 %	0	0%	0.16
FLT3–ITD	18	21.95 %	17	34%	0.16
CEBPA double-mutation	3	3.66 %	2	4%	1.00
CEBPA single-mutation	6	7.32 %	6	12%	0.37
EVI1 overexpression	4	5.13 %	4	8.33 %	0.48
*IDH1*R132 mutation	11	13.75 %	4	8.33 %	0.41
IDH2*R140 *mutation	11	13.75 %	1	2.08 %	**0.030**
*IDH2*R172 mutation	4	5%	2	4.17 %	1.00
WT1 mutation	1	1.52 %	1	2.22 %	1.00
DNMT3A mutation	26	32.91 %	19	38%	0.57
RUNX1 mutation	3	3.75 %	9	18%	**0.010**
TET2 **mutation**	8	10%	10	20%	0.12
ASXL1 mutation	5	6.85 %	3	6.38 %	1.00

Table S6: Univariate prognostic analysis for complete remission rate (CR/CRp)

	All patients				Patients with cytogenetically normal AML			
**Covariate**	**n (%)**	**OR**	**IC95%**	**P-value**	**n (%)**	**OR**	**IC95%**	**P-value**
**Karyotype**								
Favorable	9/278 (3.2)	2.30	0.28–18.73	0.69	NA	NA	NA	NA
Intermediate	186/278 (66.9)	4.14	2.29–7.50	**<0.001**	NA	NA	NA	NA
Normal karyotype	146/278 (52.5)	4.67	2.46–8.89	**<0.001**	NA	NA	NA	NA
Unfavorable	59/278 (21.2)	0.18	0.09–0.33	**<0.001**	NA	NA	NA	NA
Not available	24/278 (8.6)	0.83	0.31–2.19	0.80	NA	NA	NA	NA
**SNP-array karyotyping**								
Presence of SNP-A lesion(s)	135/248 (54.4)	0.45	024–0.86	**0.014**	50/132 (37.9)	0.57	0.19–1.74	0.39
**Molecular findings**								
MLL-PTD	6/268 (2.2)	0.54	0.10–3.05	0.61	4/142 (2.8)	0.34	0.03–3.48	0.36
NPM1 mutation	94/276 (34.1)	2.17	1.11–4.25	**0.022**	75/145 (51.7)	1.25	0.43–3.66	0.79
FLT3-TKD mutation	14/276 (5.1)	0.68	0.21–2.25	0.51	5/145 (3.4)	0.44	0.05–4.26	0.42
FLT3-ITD	49/276 (17.8)	2.81	1.6–7.45	**0.035**	36/145 (24.8)	1.36	0.36–5.12	0.76
CEBPA sm + dm	18/271 (6.6)	1.45	0.41–5.20	0.77	12/143 (8.7)	1.32	0.16–10.97	0.99
CEBPA dm	10/271 (3.7)	2.63	0.34–21.17	0.70	5/143 (3.6)	NC	NC	0.99
EVI1 overexpression	25/251 (10.0)	0.64	0.25–1.62	0.43	10/135 (7.4)	NC	NC	0.60
RUNX1 mutation	26/266 (9.8)	0.92	0.35–2.41	0.81	13/139 (9.4)	0.58	0.11–2.92	0.62
WT1 mutation	7/230 (3.0)	NC	NC	0.35	2/119 (1.7)	NC	NC	0.99
ASXL1 mutation	21/247 (8.5)	1.27	0.41–3.94	0.79	9/127 (7.1)	0.40	0.07–2.13	0.26
DNMT3A mutation	NA	NA	NA	NA	47/138 (34.1)	0.92	0.29–2.93	0.99
TET2 mutation	NA	NA	NA	NA	19/139 (13.7)	0.34	0.09–1.22	0.10
*IDH1*R132 mutation	NA	NA	NA	NA	15/137 (10.9)	0.71	0.14–3.52	0.65
*IDH2*R140 mutation	NA	NA	NA	NA	12/138 (8.7)	NC	NC	0.61
*IDH2*R172 mutation	NA	NA	NA	NA	6/137 (4.4)	0.55	0.06–5.08	0.48

Abbreviations: AML, acute myeloid leukemia; OR, odd ratio; CI, confidence interval; SNP-A, single-nucleotide polymorphism array; NA, not applicable; NC, non-convergent; PTD, partial tandem duplication; ITD, internal tandem duplication; sm, single-mutation; dm, double-mutation.

Table S7: Multivariate prognostic analysis for complete remission rate (CR/CRp) in the whole patient cohort

Covariates in final model	OR	95% CI	P-value
Presence of unfavorable karyotype	0.18	0.08–0.41	**< 0.001**
Presence of SNP-A lesion(s)	0.68	0.31–1.50	0.34
Presence of *NPM1* mutation	0.97	0.38–2.50	0.95
Presence of *FLT3*–ITD mutation	2.23	0.62–7.97	0.22
Randomization in the GO arm	2.01	0.99–4.08	0.053

Abbreviations: OR, odd ratio; CI, confidence interval; SNP-A, single-nucleotide polymorphism array; ITD, internal tandem duplication.

SUPPLEMENTARY FIGURES

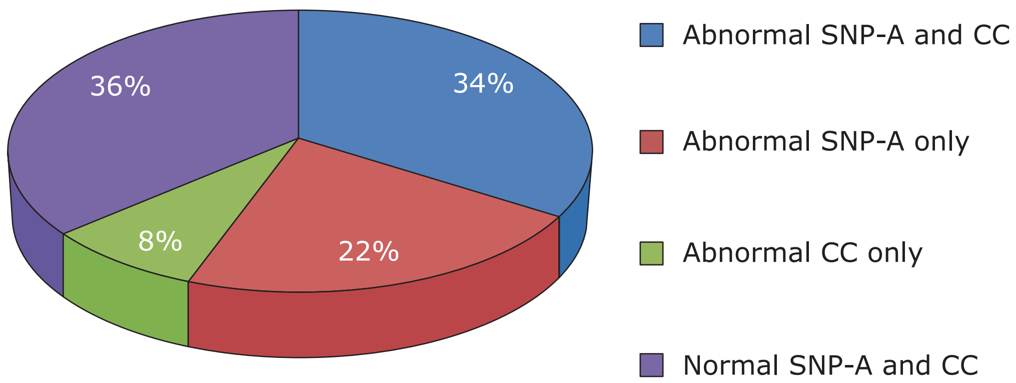

Figure S1: Pie chart representing the proportion of patients with genomic abnormalities detected by conventional cytogenetics and/or SNP array karyotyping.

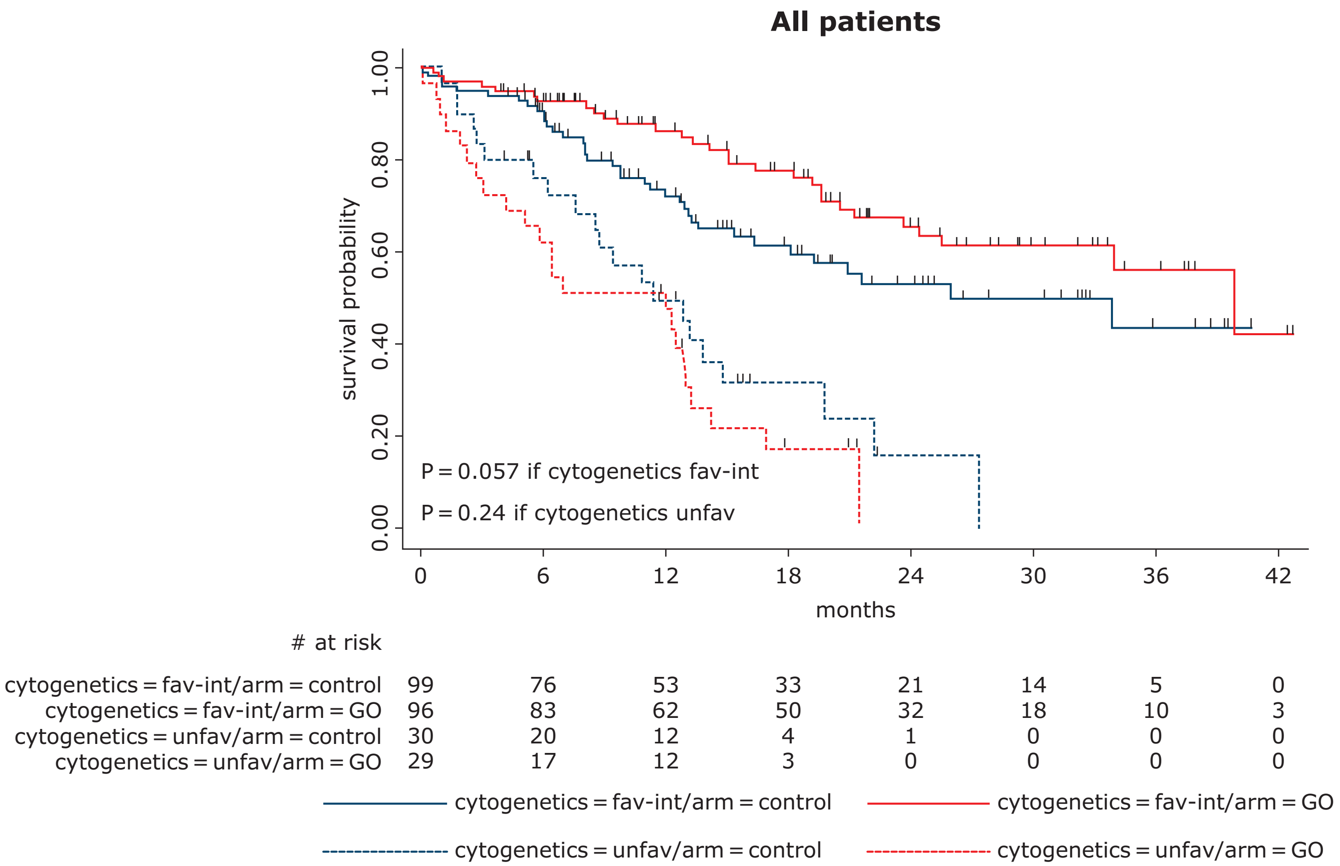

Figure S2: Kaplan-Meier estimates of overall survival according to cytogenetics (favorable or intermediate *versus* unfavorable karyotype) and treatment arm. In patients with unfavorable cytogenetics, 2–year OS was estimated at 16% (95%CI, 3–37) in the control arm *versus* 0% in the GO arm (P = 0.24 by the log-rank test). In patients with favorable/intermediate cytogenetics, 2–year OS was estimated at 53% (95%CI, 40–64) in the control arm *versus* 65% (95%CI, 53–76) in the GO arm (P = 0.057 by the log-rank test).

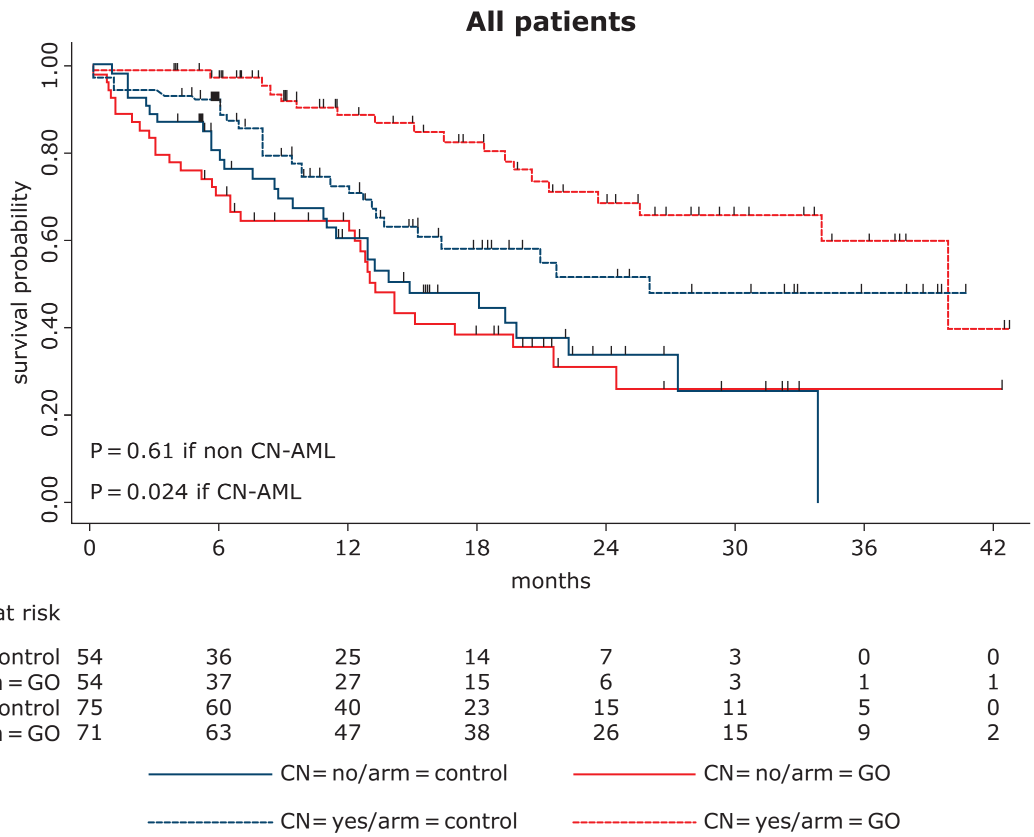

# at risk	0	6	12	18	24	30	36	42
CN = no/arm = control	54	36	25	14	7	3	0	0
CN = no/arm = GO	54	37	27	15	6	3	1	1
CN = yes/arm = control	75	60	40	23	15	11	5	0
CN = yes/arm = GO	71	63	47	38	26	15	9	2

Figure S3: Kaplan-Meier estimates of overall survival according to cytogenetics (normal karyotype *versus* abnormal karyotype) and treatment arm. In patients with cytogenetically normal (CN) AML, 2–year OS was estimated at 52% (95%CI, 36–65) in the control arm *versus* 69% (95%CI, 53–80) in the GO arm (P=0.024 by the log-rank test). In patients with abnormal cytogenetics, 2–year OS was estimated at 34% (95%CI, 19–49) in the control arm *versus* 31% (95%CI, 17–46) in the GO arm (P=0.61 by the log-rank test).

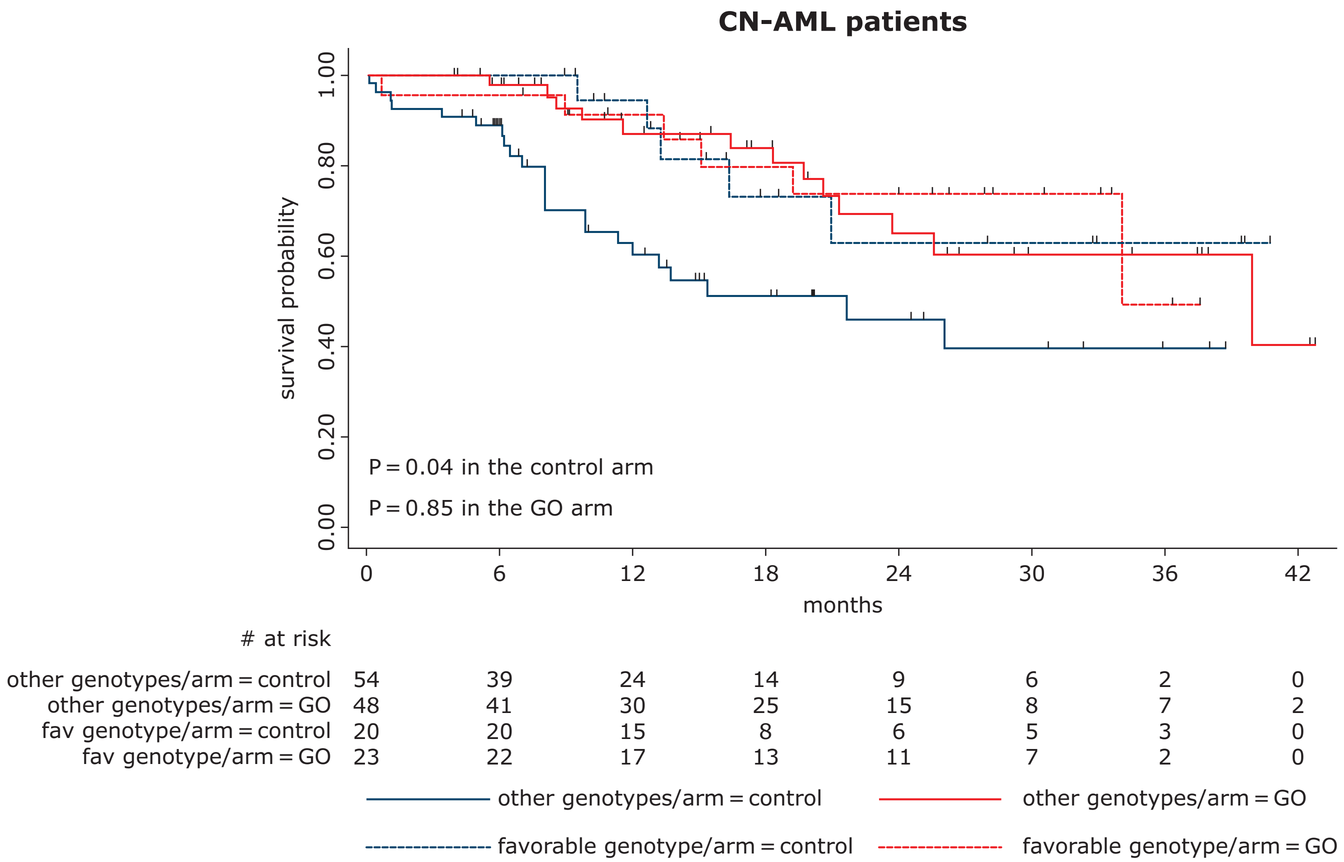

Figure S4: Kaplan-Meier estimates of overall survival according to *NPM1*/*FLT3*–ITD genotypes in cytogenetically normal AML patients. Considering the whole cohort of cytogenetically normal (CN) AML (n = 146), 2–year OS was estimated at 69% (95%CI, 50–82) in patients with favorable genotype (i.e. *NPM1* mutated/*FLT3*–ITD negative genotype) *versus* 55% (95%CI, 41–66) in patients with other genotypes (P = 0.08).

SUPPLEMENTARY REFERENCES

1. International System for Cytogenetic Nomenclature: Guidelines for Cancer Cytogenetics. In: Mitelmann F, ed. Supplement to an International System for Human Cytogenetic Nomenclature Basel, Switzerland: S. Karger; 1991:1–53.
2. Cheson BD, Bennett JM, Kopecky KJ, et al. Revised recommendations of the International Working Group for Diagnosis, Standardization of Response Criteria, Treatment Outcomes, and Reporting Standards for Therapeutic Trials in Acute Myeloid Leukemia. J Clin Oncol. 2003;21(24):4642–4649.
3. Delhommeau F, Dupont S, Della Valle V, et al. Mutation in TET2 in myeloid cancers. N Engl J Med. 2009;360(22):2289–2301.
